# Effects of a mindfulness-based program on the occupational balance and mental health of university students. Protocol for a randomized controlled trial

**DOI:** 10.1371/journal.pone.0302018

**Published:** 2024-05-02

**Authors:** Carmen Lillo-Navarro, Paula Fernández-Pires, Gemma Benavides Gil, Fermín Martínez-Zaragoza, Covadonga Chaves, Pablo Roca, Paula Peral-Gómez, María Elena González Valero, Daniel Mendialdua Canales, José Luis Poveda Alfaro, Philippe R. Goldin, Alicia Sánchez-Pérez

**Affiliations:** 1 Centre for Translational Research in Physiotherapy (CEIT), Department of Pathology and Surgery, Miguel Hernández University of Elche (UMH), Alicante, Spain; 2 B+D+b Occupational Research Group, Department of Pathology and Surgery, Miguel Hernández University of Elche (UMH), Alicante, Spain; 3 Alicante Health and Biomedical Research Institute (ISABIAL), Alicante, Spain; 4 Department of Behavioural Sciences and Health, Miguel Hernández University of Elche (UMH), Alicante, Spain; 5 Department of Personality, Assessment and Clinical Psychology, School of Psychology, Complutense University of Madrid, Madrid, Spain; 6 Faculty of Health Sciences, Universidad Villanueva, Madrid, Spain; 7 Valencian International University, Valencia, Spain; 8 Order of Interbeing, Spain; 9 Elephant Plena Center for Psychology and Training, Valencia, Spain; 10 University of California, Davis, Davis, California, United States of America; Mattu University, ETHIOPIA

## Abstract

**Objective:**

The aim is to examine whether the addition of Virtual Reality (VR) meditation training to a standard 8-week Mindfulness-Based Health Care Program (MBHC-VR) results in a significantly increased improvement in occupational, mental health, and psychological functioning versus MBHC-only in university students.

**Materials and methods:**

A randomized controlled clinical trial with three arms (MBHC, MBHC-VR, Control Group), four assessment time points (pre-intervention, inter-session, post-intervention, and 3-month follow-up), and mixed methodology will be proposed. University students (undergraduate, master, or doctoral) interested in participating and who meet the inclusion/exclusion criteria will be included over two years. Data will be collected from different ad hoc questionnaires, several standardized tests, and an Ecological Momentary Assessment. We will use R software to carry out descriptive analyses (univariate and bivariate), multilevel modeling, and structural equation models to respond to the proposed objective. The qualitative analysis will be carried out using the MAXQDA program and the technique of focus groups.

**Discussion:**

It is expected that with the proposed intervention university students will learn to relate in a healthier way with their mental processes, so as to improve their occupational balance (OB) and their psychological well-being.

**Trial registration:**

ClinicalTrials.gov Identifier NCT05929430.

## Introduction

The mental health of university students have become a major public health concern [1, 2]. Between 29% and 37% of university students report elevated stress [3, 4], which can affect OB and contribute to the development of psychopathologies such as depression and anxiety disorders [5]. Psychopathological symptoms, including stress, anxiety, sleep problems, and depression, affect the university population around the world and are associated with poor academic performance and health risk behaviors, such as substance abuse, self-harm or suicide [6–8]. In 2016, the World Health Organization conducted a study with a sample of 5,750 university students from 21 countries and concluded that 20.3% met the criteria for a mental disorder according to the DSM-IV/CIDI [9]. This situation has worsened markedly after the COVID-19 pandemic according to a recent meta-analysis that showed a prevalence of 34% depression, 32% anxiety, and 33% sleep disorders in 1,441,828 university students from 29 countries [10, 11].

All these psychological symptoms influence OB, which is defined as an individual’s subjective perception of having an adequate number and variety of occupations in daily life [12]. A good OB is related to general mental health, satisfaction with life, and low stress levels, which makes it an important aspect to consider in clinical practice [13]. Empirical evidence showed that 62% of undergraduates feel dissatisfied with their daily routine [14], and usually have a moderate OB [15].

Taking into account the personal and social burden of mental health problems and occupational imbalance, a growing number of universities currently offer services and interventions aimed at these variables, including Mindfulness Based Programs (MBPs) [16]. Mindfulness is defined as the ability to pay attention to the present moment with interest, curiosity, and acceptance [17, 18]. The practice of mindfulness may change perspective, “decentering” or “re-perception”, so that the person can perceive internal experiences with great clarity [19, 20]. This shift in perspective facilitates self-regulation, clarification of values, cognitive, emotional, and behavioral flexibility, and the ability to deal with intense emotions objectively [17]. From conceptual and neural perspectives, research has identified four underlying mechanisms of action: 1) attention regulation: sustained attention to a selected object, shifting attention to the object every time a distraction occurs; 2) sensory awareness: attention to body sensations; 3) emotion regulation, modulating emotions without judgment; and 4) change in perspective on the self, moving to the role of the observer [21].

The practice of mindfulness meditation has shown important benefits in university students. A recent meta-analysis (n = 2201; 15 countries) that examined the effect of MBPs in university students showed beneficial results when comparing to a passive control group. The effect sizes were small-to-moderate for psychological well-being, anxiety and depression symptoms, perceived well-being, rumination, and mindfulness skills [22].

However, the low level of adherence to the MBPs in university students is considered an important limitation [23]. This highlights the need to identify innovative ways to improve home practice and adherence, such as the incorporation of virtual reality (VR) [24] to facilitate meditation practice. In fact, although further studies are required, virtual reality (VR) driven mindfulness training has demonstrated greater efficacy compared to traditional mindfulness methods [25], enhancing mental health and physical well-being. It has been proven to reduce anxiety [26, 27], pain [28], emotional exhaustion [29], fatigue [30], improve sleep quality [31] or help with smoking cessation [32], among others results. Virtual reality (VR) has the potential to enable users to encounter stimuli that might otherwise remain physically or cognitively out of reach, often due to avoidance or restricted imagination [33]. VR fosters a deep sense of immersion, carrying users into the core of these environments. A recent review of studies using mindfulness based on VR demonstrated that the more immersive VR (i.e., 3-dimentional head mounted VR screen) was more effective than the less immersive one (i.e., 2-dimentional computer generated screen). VR-based mindfulness practice usually contains images of natural environments, such as landscapes, rivers, mountains, oceans, forests, trees and rocks, with background music and audio guidance [25]. A recent clinical trial showed that MBPs with VR-assisted meditation practice resulted in 95.7% adherence which is between 16% to 30% higher than MBPs without VR [5].

For this reason, our RCT study will investigate the effects of a MBPs with versus without VR-assisted meditation in university students using quantitative and qualitative methods. For the quantitative approach, we will assess positive functioning, clinical measures and ecological momentary assessment (EMA) [34], to study the relationship between individual experiences, emotions, the social context, and behaviors. For the qualitative approach, we will conduct focus groups to explore potential psychological mechanisms of change associated with the implementation of standard MBPs without and with VR-assisted meditation.

The main objective of this study is to examine the differential effects of a Mindfulness-Based Health Care Program (MBHC) without versus with VR-assisted meditation (MBHC-VR) on OB and mental health (i.e. psychological distress) in university students. Our seven objectives are:

1. To compare the training effects on occupational indicators, such as occupational balance, eating habits, sleep and physical activity.
2. To compare the training effects on mental health indicators, such as anxiety, depression, stress and burnout.
3. To compare the training effects on psychological functioning, such as emotional regulation, acceptance, mindfulness-trait, self-compassion and life satisfaction.
4. To test whether the training effects are maintained 3 months post-completion of MBHC.
5. To identify potential mechanisms of change in MBHC vs MBHC-VR.
6. To examine expectations of the participants before their participation in the program.
7. To qualitatively explore the perceptions of the participants about the program and its effects after its completion.

### Hypotheses

We hypothesized that, compared to the waiting list control group, both MBHC and MBHC-VR will improve occupational indicators (Hypothesis 1), mental health (Hypothesis 2), and psychological functioning (Hypothesis 3). We expect that, compared to MBHC, MBHC-VR will have higher adherence to home practice [5], and result in greater magnitude of change in the occupational balance, mental health and psychological functioning variables (Hypothesis 4). Furthermore, we expect that improvements will be maintained at 3-months post-intervention completion [5] (Hypothesis 5). Finally, we predict that psychological functioning variables will act as mechanisms of change of occupational balance and mental health indicators in experimental vs control condition [35] (Hypothesis 6). All hypotheses will be tested in the post-training assessment, except for hypothesis 5, which will be tested in the follow-up.

## Material and methods

### Study design and setting

A randomized controlled clinical trial (RCT) with three arms and four assessment timepoints (i.e., pre-intervention, inter-session, post-intervention, and 3-month follow-up) using qualitative and quantitative methods. The three arms include an adapted MBHC [36], MBHC plus VR-assisted meditation (MBHC-VR), and a waiting list control group (WL). The study protocol has followed the SPIRIT guidelines [37] (S2 Appendix) and RCT was conducted following the recommendations of the Consolidated Standards of Reporting Trials (CONSORT) guidelines [38].

The clinical trial is based in the university of Miguel Hernández University of Elche which has a population of about 15000 students. This population is generally representative of university student populations in Spain. The duration of study participation for each student is five months in order to incorporate potential differences between the first and second semester of the academic year.

### Ethical consideration and declaration

The protocol of this clinical trial was approved by the Research Ethics and Integrity Committee of the Miguel Hernández University (DPC.ASP.01.22) and by Committee of Ethics of the Research with Medicines of the of the Elche General University Hospital (PI 132/2022) (S3 and S4 Appendices). Furthermore, the research was registered with ClinicalTrials.gov (ClinicalTrials.gov Identifier: NCT05929430) and will be performed in accordance with the Declaration of Helsinki as well as the Organic Law 3/2018 (December 5) on the Protection of Personal Data and Guarantee of Digital Rights. Data confidentiality is warranted during the whole research process (i.e., data collection, data cleaning and dissemination of research results).

Participation in the clinical trial will be voluntary, and participants will provide informed consent prior to their inclusion in the study. Data confidentiality will be protected during the research process (i.e., data collection, data cleaning, and dissemination of research results).

### Sample size estimation

G *Power (v. 3.1) was used to estimate the sample size needed to test hypotheses 1, 2, 3 and 5. A mixed model using treatment as a between-subjects factor (MBHC, MBHC-VR, and WL) and time as a within-subjects factor (pre, inter, post, and follow-up) will be tested. Interaction effect was powered. The sample size was estimated taking as a reference a small difference in perceived stress between MBHC with vs without VR-assisted meditation in university students [5]. With an alpha criterion of .05 and a small effect size (Cohen’s d^2^ = .08), to achieve a minimum of 80% power, a minimum sample size of 174 participants will be needed. Assuming an attrition rate, based on complete dropout of approximately 20% [5], the final sample needed will include at least 210 university students (i.e., 70 participants in each of three groups).

### Participants

Participants will be bachelor degree, masters, or doctorate students at Miguel Hernández University of Elche. Recruitment will be carried out over two academic years. Invitations to participate will be disseminated by institutional mailings, university’s official website, informative posters, and dissemination talks. Students interested in participating will, voluntarily pre-enroll by completing a brief online questionnaire on contact information, eligibility criteria, previous experience in meditation, and program expectations.

Inclusion criteria include: a) being 18 years old or older; b) enrolled in a bachelor degree, master or doctorate degree program at Miguel Hernández University of Elche; c) speak Spanish fluently; d) signing the Informed Consent form; and g) having internet access from a computer or smartphone to complete the online assessments and formal practices during the study. Furthermore, participants will be excluded if they: a) report a severe mental disorder in active phase diagnosed by a health professional (e.g., schizophrenia, bipolar disorder, substance abuse/dependence); b) are under the influence of alcohol or other drugs during sessions and/or assessments (determined by the program instructor); and c) participate in another standardized meditation program during the study period. Should any participants require psychological or psychiatric treatment during the study, they will be referred to specialized care services.

### Procedure

When students who wish to participate submit the form indicating their interest, eligible students will be interviewed by telephone to confirm compliance with the eligibility criteria and will be informed orally and in writing (via e-mail) about the research. If they decide to participate, they will be administered the on-line pre-intervention assessment one week before starting the program and once they give their informed consent. Ineligible students will not be allowed to enroll in the study.

Participants will not pay for MBHC training and will be rewarded with 1 European Credit Transfer System (ECTS) for their participation at the end of the study, when they have completed the last follow-up assessment.

### Randomization, allocation and blinding

Participants who satisfy eligibility criteria will be randomly assigned to one of the three intervention groups in a ratio of 1:1:1 using the randomize R package in R [39]. Randomization will be performed after the baseline assessment to preserve allocation concealment.

**Fig 1:**
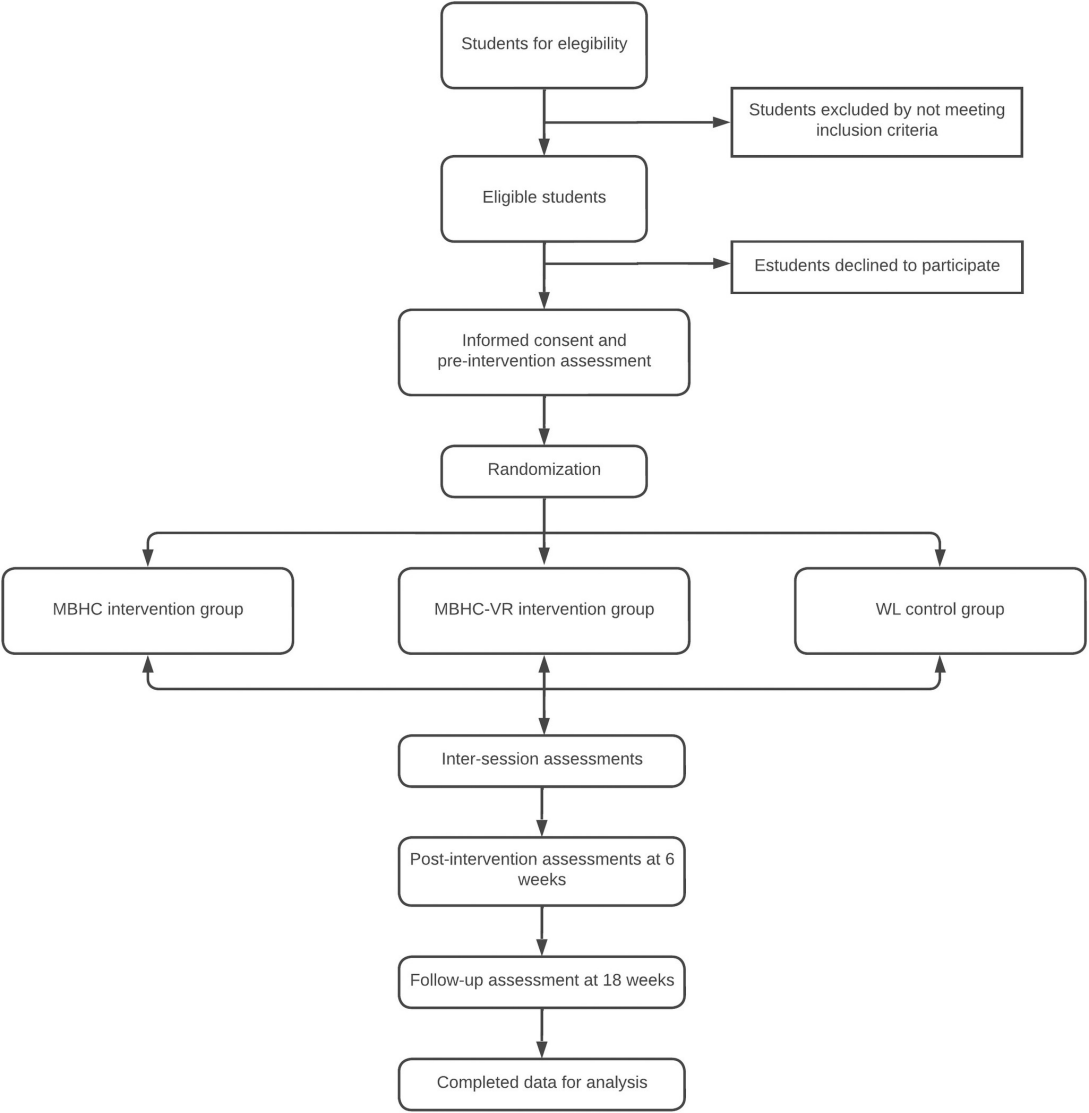
Flowchart of the study procedure. Abbreviations: MBHC: Mindfulness-Based Health Care; MBHC-VR: Mindfulness-Based Health Care with virtual reality; WL: waiting list.

The participants will be randomly assigned to the MBHC, MBHC-VR or WL and informed by telephone one week before starting intervention or WL groups. Fig 1 summarizes the flowchart of the study.

To limit potential selection bias, members of the research team who are not involved in the assessments and implementation of the intervention will be responsible for the random sequence and data analysis.

### Intervention groups

#### Mindfulness-Based Health Care Program (MBHC)

The MBHC program is adapted for university students from the Mindfulness-Based Stress Reduction (MBSR) program [40]. MBSR is an 8-week evidence-based program that cultivates mindfulness of present-moment and a nonreactive, nonjudgmental attitude toward the experience. This version of the MBHC program [36] will be offered in-person and has a similar structure to the MBSR program but is focused for university students and introduces elements related to healthy habits and lifestyle. Features of MBHC that differ from MBSR will include: a) 6-weeks instead of 8-weeks of group instruction; b) weekly 1.5 hours sessions instead of 2.5–3 hours; and c) specific formal and informal practices aimed at cultivating healthy habits and lifestyle (e.g., activities of daily living such as feeding, bathing or showering and other occupations such as communication management, health management, etc. [41]; and prosocial components (e.g., kindness and compassion).

Core components of MBHC include: a) paying attention to the present moment; b) cultivating acceptance and openness to the present experience, without resistance and judgment; c) developing and strengthening prosocial qualities, such as kindness and compassion; d) enhancing deep self-inquiry by examining subjective experience of thoughts, feelings, and sensations; and e) promoting healthy habits and occupations. We will also include person-centered facilitation techniques, which include: 1) Unconditional positive regard; 2) Empathy; 3) Authenticity in interactions; 4) Client-centered focus; 5) Non-directive approach; 6) Self-actualisation; 7) Reflection and clarification; 8) Non-judgmental listening; 9) Holistic approach; and 10) Emphasis on the “Here and Now”; [42] and non-violent communication practices (Observation of the situation, Recognition of feelings, Identification of needs, Formulation of clear requests) [43] to support a conducive learning environment and facilitate communication. All sessions will include mindful movement, formal and informal meditation practices, sharing personal experiences and thoughts, and exercises to do at home. At the end of each session, participants will receive a set of pre-recorded audio files and printed material to support their daily practices. The structure of the MBHC program sessions is included in *S1 Appendix*. Additionally, as an example, the material of the first session is available on the web page of the B+D+b Occupational Research Group of the Miguel Hernández University.

MBHC and MBHC-VR programs will be conducted by two instructors, both with over 25 years of experience in Tibetan, Theravada and Zen meditation; experience in retreats of weeks and months in the West and Sri Lanka, and with more than 15 and 8 years of experience, respectively, facilitating mindfulness-based programs. The program has been previously implemented at Miguel Hernández University of Elche since 2016, involving university students.

#### Mindfulness-Based Health Care program with virtual reality (MBHC-VR)

Mindfulness practice with VR is an innovative way to use technology to enhance the meditation experience. This technique combines audio guided mindfulness practice (S1 Appendix) with immersion in a virtual environment that can have multiple benefits, including a higher rate of training adherence [5]. The content and objectives of MBHC and MBHC-VR programs are the same. The primary difference is that some practices (S1 Appendix) of each session will be done, within the in-person session and at home, using VR. The VR kit consists of Meta Quest 2 VR goggles and access to 360° mindfulness videos. To perform the VR practices at home, participants will be provided with VR glasses.

#### Waiting list control group (WL)

Participants in the WL control group will not receive any intervention during the study. However, for ethical reasons, at the end of the 3-month follow-up assessment, participants of this WL control group will be invited to participate in the MBHC program free of charge.

### Measures

At baseline (i.e., pre-training assessment), we will collect demographic information (age, sex, education level, marital status, work status and experience in meditation), qualitative questions about expectations regarding the program, and standardized self-report questionnaires assessing occupational balance (OBQ-X), anxiety (DASS-21), depression (DASS-21), stress (DASS-21; EMA), academic stress (SISCO Inventory), burnout (MBI-SS), emotional regulation (DERS; EMA), mindfulness (FFMQ-SF; EMA), self-compassion (SCS-SF; EMA), life satisfaction (SLS), psychological flexibility (AAQ-II; EMA), social desirability (M-C SDS), diet (PREDIMED), physical activity (IPAQ-SF), and sleep (ISI).

During the 6-weeks of MBHC or WL (i.e., inter-session assessment), information about perceived stress, emotional regulation, mindfulness, self-compassion, psychological flexibility, and volition will be collected daily by Ecological Momentary Assessment (EMA) [34]. To carry out the momentary measurements, we will use the Google Forms tool to send via smartphone daily requests for information, that will be sent daily once a day, between 19:00 to 20:00 PM, to the participants. The EMA assessment form will include questions in both text format and visual analog scales for response. A research team member will supervise the EMA and will serve as a contact and reference via telephone to respond to questions and resolve any technical difficulties.

At immediately post-MBHC or WL, we will collect self-report responses using the same assessment tools used in the baseline assessment. At 18 weeks after the baseline assessment (i.e., 3-months post-MBHC or WL follow-up assessment) the same self-report set of questionnaires (minus social desirability and mental imagery) will be administered.

### Quantitative assessment

Information about demographics, health issues, and previous meditation experience will be collected at baseline. Table 1 shows all the measures that will be used in each assessment timepoint.

**Table 1:** Summary of the quantitative data collection for the research.

Outcome Measures	Pre-intervention	Inter-session^a^	Post-intervention	3-month follow-up	Measurement Method
**Main outcome**					
Occupational Balance	X		X	X	OBQ-E
Anxiety	X		X	X	DASS-21
Depression	X		X	X	DASS-21
Stress	X	X	X	X	DASS-21; EMA
Academic stress	X		X	X	SISCO Inventory
Burnout	X		X	X	MBI-SS
**Secondary outcome**					
Emotional dysregulation	X	X	X	X	DERS; EMA
Mindfulness	X	X	X	X	FFMQ-SF; EMA
Self-compassion	X	X	X	X	SCS-SF; EMA
Life satisfaction	X		X	X	SLS
Psychological flexibility	X	X	X	X	AAQ-II; EMA
Social desirability	X		X		M-C SDS
Adherence to the Mediterranean Diet	X		X	X	PREDIMED
Physical activity	X		X	X	IPAQ-SF
Sleep	X		X	X	ISI
Mental representations	X		X		Psi-Q
Volition		X			EMA
Adherence		X		X	EMA; ad hoc questionnaire
**Sociodemographics**					
Age (in years)	X				ad hoc questionnaire
Sex	X				ad hoc questionnaire
Education level	X				ad hoc questionnaire
Marital status	X				ad hoc questionnaire
Work status	X				ad hoc questionnaire
Experience in mindfulness	X				ad hoc questionnaire

Abbreviations: OBQ-E Occupational Balance Questionnaire; DASS-21, Anxiety, depression and stress scale; MBI-SS, Maslach Burnout Inventory-Student Survey Questionnaire; DERS, Emotional Regulation Difficulties Scale; FFMQ-SF, The Five Facets of Mindfulness Questionnaire short FORM; SCS-SF, Self-Compassion Scale short form; SLS, Life Satisfaction Scale; AAQ-II, Acceptance and Action Questionnaire II; M-C SDS, Marlowe-Crowne Social Desirability Scale short form; Physical Activity Questionnaire short form (IPAQ-SF); Insomnia Severity Index (ISI); Plymouth Sensory Imagery Questionnaire (Psi-Q). ^a^ Inter-session measured with the Ecological Momentary Assessment (EMA).

*Main outcomes*. *Occupational Balance Questionnaire* (OBQ) [44] is a 13-item tool scored on a 6-point Likert-type response scale, ranging from 0 (strongly disagree) to 5 (strongly agree), with a total score ranging from 0 to 65, where a higher score indicates greater OB. The internal consistency of this instrument is 0.87.

*Depression*, *Anxiety and Stress Scale* (DASS-21) [45] measure psychological distress with 7 items for each subscale: depression, anxiety and stress. Participants rate the degree to which each statement has happened to them in the past week on a scale from 0 (has not happened to me) to 3 (has happened to me a lot or most of the time). The internal consistency of this assessment tool is 0.90.

Academic stress will be measured using the *SISCO Inventory* [46] which consists of 31 items distributed into: 1 filter item (yes-no), 1 item on a Likert-type scale from 1 (a little) to 5 (a lot), 8 items on a Likert-type scale from 1 (never) to 5 (always) that allow identifying stressful stimuli, 15 items on a Likert-type scale from 1 (never) to 5 (always) that allow identifying stressful stimuli divided into physical, psychological and behavioural reactions, and 6 items on a Likert-type scale from 1 (never) to 5 (always) allowing to identify the frequency of use of coping strategies. Its internal consistency is 0.90.

*Maslach Burnout Inventory-Student Survey Questionnaire* (MBI-SS) [47] contains 15 items grouped into 3 subscales: Emotional Exhaustion (5 items), Cynicism (4 items), and Academic Efficacy (6 items). These items are scored on a scale from 0 (never/never) to 6 (always/every day). High scores on burnout and cynicism and low scores on academic efficacy are indicative of burnout. The internal consistency of this instrument is 0.84.

*Secondary outcomes*. *Difficulties in Emotional Regulation Scale* (DERS) [48, 49] contains 28 items grouped into 5 subscales: emotional lack of control, life interference, lack of emotional attention, emotional confusion, and emotional rejection. These items are scored on a Likert-type scale from 1 (almost never) to 5 (almost always). Higher scores indicate greater difficulties in emotion regulation. Its internal consistency is 0.93.

*Acceptance and Action Questionnaire II* (AAQ-II) [50] measures psychological flexibility using 7 items with a 7-point Likert-type scale from 1 (never true) to 7 (always true). Scores range from 7 to 49. Higher scores indicate a tendency to act on the need to control or avoid aversive thoughts, memories, or feelings. The internal consistency of this instrument is 0.88.

*Five Facets of Mindfulness Questionnaire Short-Form* (FFMQ-SF) [51] contains 24 items that assess the five facets of mindfulness: observing, describing, acting with awareness, non-judgement of inner experience, and non-reactivity to inner experience. It is scored on a Likert-type scale from 1 (never or very rarely true) to 5 (very often or always true). Higher scores indicate a greater capacity for mindfulness. The internal consistency of this assessment tool is 0.86.

*Self-Compassion Scale* short form (SCS-SF) [52] consists of 12 items assessing 6 factors: Self-Kindness, Self-Judgment, Common Humanity, Isolation, Mindfulness, and Over-identification. Each item can be rated on a Likert-type scale from 1 (almost never) to 5 (almost always). Higher scores indicate greater self-compassion. The internal consistency of this instrument is 0.85.

*Satisfaction with Life Scale* (SLS) [53] consists of 5 items with a 7-point Likert-type scale (from 1, strongly disagree; to 7, strongly agree). The score ranges from 5 to 35, with higher scores indicating greater satisfaction. The internal consistency of this instrument is 0.88.

*Marlowe-Crowne Social Desirability Scale* short form (M-C SDS Scale) [54] consists of 18 items, 13 items reflecting very frequent undesirable behaviours and traits, and other 5 items reflecting infrequent socially desirable behaviours and traits. The person being assessed must indicate whether they are true or false with regard to him/herself. The sum of the scores gives a total score between 0 and 18, where a higher score indicates a higher social desirability, understood as a response bias or personality trait. It internal consistency is 0.76.

*Mediterranean Diet Adherence Test* [55] is a brief dietary assessment consisting of 14 questions that evaluates adherence to the Mediterranean Diet pattern. This questionnaire scores one point per question, with a score of <9 meaning low adherence and a score of > = 9 meaning good adherence.

*Insomnia Severity Index* (ISI) [56] is a 5-item self-report instrument that assesses the severity of the sleep problem, the degree of dissatisfaction and the impact on quality of life. Scores range from 0 (best score) to 28 (worst score). The internal consistency of this instrument is 0.82.

*International Physical Activity Questionnaire* short form (IPAQ-SF) [57] is a self-administered questionnaire consisting of 7 items that assesses the time spent being physically active in the last 7 days. The internal consistency of this assessment tool is 0.337.

*Plymouth Sensory Imagery Questionnaire* (Psi-Q) [58] measures vividness of mental imagery and consists of 21 items measuring 7 sensory modalities: vision, sound, smell, taste, touch, bodily sensation and emotions. The questionnaire has a Likert-type scale between 1 (no imagery) and 7 (perfectly clear and as vivid as the actual experience). The internal consistency of this instrument is 0.88.

*Intervention adherence*. During weeks 1–6 of MBHC program, adherence will be recorded by means of: 1) class attendance, 2) number of minutes practicing mindfulness each day (question number 11 of the EMA). In the follow-up period (weeks 7–18) participants will receive a weekly survey asking for the frequency (number of days per week) and duration of mindfulness practice (minutes) during the last week [59].

*Ecological momentary assessment*. This evaluation "at the moment" and "in the current context" presents multiple methodological advantages compared to traditional data collection systems: 1) it reduces memory bias by obtaining current or recent experience information; 2) allows the collection of information in the environment of the subject, increasing compliance and reliability; 3) and allows the detection of variations over time and factors that influence the evolution of the participants [34]. Our EMA will include 11 questions (Table 2) assessing occupation (question 1), volition (question 2), mindfulness state (question 3), well-being state (questions 4 and 9), positive and negative affect of high and low arousal (questions 5 and 6), state stress (question 7), self-compassion (question 8), recovery experience (question 10) and monitoring of daily mindfulness practice (question 11). Questions 2 to 11 will be answered using a visual analogue scale from 0 to 10 points.

**Table 2:** Questions included on Ecological Momentary Assessment (EMA).

1. What are you doing right now?
2. This activity is motivating for me
3. I was thinking about something other than what I was doing right now
4. What is my mood right now?
5. I have felt happy and in a good mood while doing that activity
6. I have felt calm and relaxed while doing that activity
7. What is my stress level right now?
8. While doing the activity, have I given to myself the care and kindness that I need?
9. What is my level of happiness right now?
10. In the last 24 hours I have carried out some recovery activity/experience (I have slept well, I have played sports, leisure activities, etc.)
11. In the last 24 hours, how many minutes have I spent practicing mindfulness?

#### Qualitative assessment

We will conduct focus groups with all participants in the MBHC and MBHC+VR arms of the trial.

During the focus groups, we will ask questions to elicit a deeper understanding of participants’ experiences, considering that group interactions can trigger responses and generate insights that may not emerge during individual interviews. Each focus group will occur within 2 weeks of MBHC completion and will be carried out by two researchers (a moderator and an assistant who will be in charge of logistical issues, and will take written notes of aspects that occur during the focus groups).

The participants will be distributed in groups of between 3 and 8 participants, segmented by type of intervention received during the trial, and by gender, since it has been described that carrying out focus groups with people of the same gender generates greater quantity and depth in the answers. Escobar and Bonilla-Jimenez recommend having a homogeneous group if the study aims to gather information that comes from shared experiences [60].

This technique will be carried out by means of a flexible script of open questions (Table 3) by experts in the subject of study, but unknown by the participating subjects, and will be recorded by means of voice recording devices. Focus groups will continue until no new issues are identified, which will suggest data saturation. The information obtained in these focus groups will be organized into meaningful patterns in order to draw conclusions.

**Table 3:** Thematic guide for focus group discussions.

How did you feel during the mindfulness sessions? And after the sessions, have you noticed any changes?
Have you noticed any positive effects that you associate with carrying out the mindfulness program? Have you noticed any negative effects that you associate with carrying out the mindfulness program or the use of virtual reality?
What problems have you had to attend the sessions? And to do it at home?
What aspects make it easier for you to attend the sessions? What aspects make it easier for you to do it at home?
Are you satisfied with the program? What aspects did you like the most? Which ones did you like the least, or what would you change about the program to improve it?
Which of your expectations have been met? Which ones don’t?

### Adverse events and safety considerations

The scientific literature on meditation-associated adverse events [MRAEs] is sparse and contradictory. However, some recent studies have suggested that MRAEs do occur in these meditation-based interventions, including those that focus on mindfulness [61–63]. According to a recent meta-analysis [64], the prevalence of MRAEs in experimental studies is 3.7% (95% CI = 0.02–0.05). The most frequent MRAEs were anxiety, depression and cognitive abnormalities (e.g. thought disorganization), stress and hallucinations and, normally, they appeared during or immediately after the practice [64].

On the other hand, regarding VR, scientific evidence shows disorientation followed by nausea and oculomotor disturbances as the most frequent negative effects, although their magnitude seems to be conditioned by factors such as the socio-demographic characteristics of the users, the VR content (neutral versus exciting) and the exposure time (adaptation after repeated exposures and greater orientation in exposures equal to or greater than 10 minutes) [65].

In this clinical trial, the participants will be encouraged to report any adverse events occurring during practice as soon as possible by WhatsApp or in-person during class. Any adverse events will be reported by the principal investigator to the Research Ethics and Integrity Committee of the Miguel Hernández University using an adverse event report. Additionally, should any participants require psychological or psychiatric treatment during the study, they will be excluded and referred to specialized care services.

### Data analysis

#### Quantitative data analysis plan

The data analysis plan will be conducted following four steps. R Software [66] will be used for all data cleaning and statistical tests with a significance level of alpha < .05.

*Data preprocessing*. Demographics and clinical records will be described at baseline by means of frequencies (percentages) or means (SD). Independent *t*-test for continuous data and chi-square test (*χ*^2^) for categorical data will be conducted to confirm that there were no demographic or clinical differences between groups at baseline. Following the CONSORT guidelines [38], Intention-To-Treat analysis will be performed using Maximum Likelihood estimation via Expectation Maximization imputation [67]. The procedure suggested by Hair et al [68] will be followed to handle missing data: 1) the types of missing data and percentage of missing will be analyzed to ensure that none exceed the recommended thresholds; 2) Little’s MCAR (Missing Completely At Random) test will be used to assess whether the missing data is completely random; and 3) sensitivity analysis will be performed to determine the impact of missing data on the results of a study, comparing the main outcomes between completers and imputed values.

*Multilevel Modelling (MLM)*. First, efficacy of MBHC and MBHC-VR compared to WL control condition will be analyzed by using MLM. Multilevel analysis enables the control of variance associated with random factors without the need for data aggregation. Variance across participants, instructor of each group, and subgroup of delivery (i.e., edition) will be modeled as random effects. Group (i.e., MBHC, MBHC-VR, and WL) and time (i.e., pre-intervention, inter-session, post-intervention, and 3-month follow-up) will be modeled as fixed effects. Analyses will be conducted via restricted maximum likelihood estimation (REML), which provides a less biased estimate of variance components with small sample sizes [69]. The group x time interaction will be used to study the trajectories of each group throughout the intervention and to determine whether the between-group differences are maintained over time.

P-values will be obtained through likelihood ratio tests comparing the full model (including the effect in question) with the model that excludes this effect. Z-values will be obtained to test the significance for fixed effects (estimates and standard errors data in the tables). Trends of the longitudinal data will be studied by testing the linear trend of the model for all the outcomes. Interaction effects between time and predictors will be performed. To estimate the magnitude of between-group differences at post-intervention and 3-month follow-up, effect sizes (Cohen’s *d*) will be calculated by dividing the differences in means by the pooled standard deviation (SD). The Cohen convention was used to interpret effect sizes, where effect sizes of 0.20 are categorized as small, effect sizes of 0.50 are classified as medium, and effect sizes greater than or equal to 0.80 are considered large.

Model comparison approach will follow Bliese and Ployhart [70] and Bliese [71] guidelines. The process begins by examining the nature of the outcome. To test the significance of person effects, a likelihood ratio test will be conducted to compare the null multilevel model (i.e., unconditional model) with a null single-level model, testing the null hypothesis that there are no group differences. The second step involves estimating the intraclass correlation coefficient (ICC) to calculate the between/within variation ratio. Model fit will be assessed using chi-square tests on the log-likelihood values and the Akaike’s information criterion (AIC) [72] relative goodness of fit index. According to the change in these fit indexes, the model with the last significant change will be chosen for each analysis.

MLM will be performed using the lme4 R package [73]. P-values of the lme4 outputs will be obtained by the lmerTest R package [74]. The Intraclass Correlation Coefficient (ICC) will be calculated by the sjstats R package [75]. Graphic data processing will be performed by the ggplot2 R package [76]. Visual inspection of q-q plots by the car R package [77] will reveal any deviations from homoscedasticity or normality in the dependent variables. In order to calculate multiple comparisons, Tukey/Bonferroni correction tests will be performed.

*Structural equation model (SEM)*. We will use the lavaan R package to implement structural equation models (SEM) by to investigate whether there are any significant mediators of training outcome. Results will be reported following the recommendations in Raykov et al. [78]. Cases with missing values and outliers will be detected using Mahalanobis distance. Multivariate and univariate normality assumptions will be tested using Mardia’s test and Kolmogorov-Smirnov’s test with the MVN R package [79]. If non-normality is found, Satorra-Bentler scale test and standard bootstrapping will be used. Model fit will be assessed using several criteria [80]: 1) a ratio of *χ*^2^*/gl<2* suggests an acceptable fit; 2) A RMSEA size below .06 suggests a well-fitting model; 3) a comparative fit index (CFI) above .95 indicates a good fit; 4) a SRMR of less than .09 also indicates a good fit; and 5) The chi-square statistic provides a conventional measure of model fit.

#### Qualitative data analysis plan

Data analysis will be carried out following the modified grounded theory approach [81]. Interviews will be transcribed verbatim and checked for accuracy. They will be imported into the MAXQDA program [82], from which the analysis will be performed. A thematic index (coding) will be built, and it will be applied independently to the first transcripts by three researchers. The following process will be followed: 1) selection of the most significant phrases; 2) initial grouping of significant phrases into categories and themes; 3) creation of identification codes for categories and themes; 4) validation of the categories and redefinition where appropriate.

Subsequently, all researchers will again check the adequacy of the interpretation of data to ensure that data allocation is systematic and verifiable. Finally, a conceptual map will be made, and the data will be interpreted, explaining the association patterns.

## Discussion

The present randomized clinical trial has been designed to evaluate and compare the effects of MBHC and a MBHC-VR on university students’ OB and mental health. For this purpose, a wide variety of indicators related to occupation (OB, eating and sleep habits, and physical activity), mental health (anxiety, depression, stress, academic stress and burnout), and psychological functioning (emotional regulation, psychological flexibility, acceptance, mindfulness-trait, self-compassion, and life satisfaction) will be evaluated in three randomized groups (i.e., MBHC intervention group, MBHC-VR intervention group, and WL control group) and four moments (i.e., pre-intervention, inter-session, post-intervention, and 3-month follow-up). The analysis of the variables will be carried out through retrospective questionnaires, EMA, and focus groups.

Although there are previous MBPs targeting university students, to our knowledge, this is the first time that mindfulness supported by VR will be explored by mixed methodology and EMA. In addition, an important difference is that the present study considers the impact on occupation, that is, how mindfulness affects the activities of daily life of the students.

Nevertheless, this study presents several limitations that should be considered. Firstly, one of the main difficulties in planning this clinical trial has been the selection of meditations for the MBHC-VR group. Several apps and programs have been developed for the application of the MBIs with VR, but it has not been possible to find any that are comparable, for comparative purposes, with the meditations programmed for the MBHC group. That is why it was finally decided to maintain the same verbal content of all the meditations in both groups, accompanying them with 360° images and evocative sounds of the themes of each meditation for the MBHC-VR group. Secondly, recruiting university students as research participants is a great challenge [83]. Some factors that influence their participation are understanding the benefits of participation, having incentives (monetary or not), receiving course credits, and facilitating logistics, among others. Therefore, several dissemination strategies (such as informative posters and dissemination talks) and granting 1 European Credit Transfer System (ECTS) for participation are proposed in this research. Lastly, it is important to take into account the low adherence that tends to be found in the MBIs [84–86]. This problem can be solved through adequate screening of the participants, daily monitoring through EMA, reminders of face-to-face sessions, and the implementation of VR. In addition, although a probable dropout rate was calculated when designing this clinical trial, some complete dropouts may occur during the study, compromising the validity of the results. One strategy to reduce the dropout rate is to call students who miss a session, to inquire about the reason for their absence. Participants who do not attend the 6-week post-intervention assessment will be called back for the 28-week follow-up assessment in order to address the problem of missing data. As for the control group, it could happen that some of the participants practiced relaxation and/or yoga techniques, etc., which could generate intervention biases. However, information on any activity regularly practiced by this group during free time will be collected to control the probable influence of these variables.

This study also presents several strengths. The study design is based on previous similar clinical trials, although some improvements were introduced. Since mindfulness itself is a complex construct to evaluate, different questionnaires that evaluate processes related to this construct will be used (FFMQ-SF, SCS-SF, DERS, AAQ-II), in order to obtain more complete data. A social desirability scale (M-C SDS) is also included, since in the psychometric assessment of mindfulness this type of effect may occur (participants may try to show a more positive or more "mindful" image of themselves, even when this self-representation does not reflect their true reality) [87]. Besides, the EMA has been added to the traditional retrospective evaluation, a methodology that evaluates the change on a day-to-day basis, and at the very moment in which the events occur, which improves the ecological validity and eliminates the possible memory bias associated with the retrospective assessment instruments. On the other hand, the qualitative methodology, based on focus groups, allows a better understanding of the change process, as well as knowing what the experiences, difficulties, preferences, and satisfaction after the programs. This mixed methodology can provide more extensive and in-depth information on the MBIs, as it will help to know not only the efficacy and the long-term effects of the interventions, but also their acceptability. Feedback from participants will allow the research team to improve the programs in order to better meet the needs of the target population.

Furthermore, this study addresses an occupational engagement-based perspective. That is, variables related to daily occupations are included, with the intention of improving university students’ meaningful and balanced participation in daily occupations. To our knowledge, research on mindfulness and OB in university students is limited.

The gender perspective is also considered. In this sense, not only the biological sex of the participants will be collected, but also the gender with which they identify. Both variables will be taken into account in the phase of statistical analysis of the data, in order to verify their relationships with all the variables evaluated and the effect of the MBIs on them.

In this study, special attention is also paid to the possibility of adverse effects associated with the practice of meditation, as evidenced by previous studies [64, 88, 89]. In anticipation of this, the participants will communicate to the research team any difficulty or symptomatology that may arise, in order to attend to and solve these events as quickly as possible and, if necessary, refer the participant to the specialized psychological services available at the university. Likewise, participants will also report possible adverse effects during and/or after VR exposure. In the MBHC-VR group of this research, VR with neutral and pleasant contents will be used in all sessions for a time between 10 and 15 minutes, favoring adaptation to the exposure and minimizing possible adverse effects.

It is also worth mentioning that the programs will be implemented by a multidisciplinary professional group with extensive experience in university education and highly qualified in MBIs. In this sense, the team is made up of occupational therapists, psychologists, physiotherapists, mindfulness experts and university professors’ experts in teaching and research.

In terms of feasibility, face-to-face interventions will be carried out in groups of about 15 people, who can be attended simultaneously, thus being a low-cost community mindfulness program for university students. If the MBHC programs designed for this study are effective, one of the advantages is that their applicability can be extended to other universities. Also, the materials and procedures will be available to the educational and scientific community. Thus, the range of effective interventions available to address university students’ mental health will be expanded.

The present trial can also contribute to expanding knowledge on personalized treatments. Baseline characteristics of individuals have started to be used as predictive factors of differential response to intervention strategies. This study will allow identifying individuals within the included sample that will benefit more from utilizing one condition relative to the other. It will also allow to establish moderating mechanisms of efficacy.

In short, and from the point of view of clinical practice, it is expected that with the proposed intervention university students will learn to relate in a healthier way with their mental processes, so as to improve their OB and their psychological well-being.

## Supporting information

S1 Appendix Structure of sessions in the mindfulness-based health care program. (DOCX)

S2 Appendix SPIRIT checklist. (DOCX)

S3 Appendix Research project submitted to the ethics committee (English). (DOCX)

S4 Appendix Research project submitted to the ethics committee (Original). (DOCX)
