# Supplementary material for: Effects of a mindfulness-based program on the occupational balance and mental health of university students. Protocol for a randomized controlled trial
Source: PLoS One. 2024 May 2;19(5):e0302018. doi: 10.1371/journal.pone.0302018 (PMC11065289; doi:10.1371/journal.pone.0302018)
Supplement: S1 Appendix — (DOCX) [file pone.0302018.s001.docx]

| **Appendix 1. Structure of sessions in the Mindfulness-Based Health Care program.** | | | |
| --- | --- | --- | --- |
| **Session** | **Main Topic** | **Content** | **Home Practice Exercises** |
| 1 | Cultivating presence | - Breathing exercises and conscious posture. Adapted from Brief grounding exercise*(1) - Visualization meditation. Adapted from Pebble exercise* (1) - Written exercise: perception of current personal situation and expectations of the course - Introductions and orientation of the course - Mindful eating exercise: eating a piece of fruit (2)   - Sharing experiences with the group   - Short practice exercise: “The pause” (3)   - Meditation Total relaxation* (2) - Sharing experiences with the group. - Instructions on home practice exercises. | - - Meditation: “Total relaxation”(2), once a day*   - Short practice exercise: “The Pause” (3), 3 times a day   - Mindfulness in an activity of daily life, once a day |
| 2 | Awakening joy | - Mindful movements* - Meditation: “The Inner Smile” (4) - Sharing experiences (including both session and home practice exercises) with the group - Short practice exercise: “10 Breaths for Happiness”* (5) - Calendar of pleasant events (6) - Meditation: “Total Relaxation” (body scan)* (2) - Sharing experiences with the group. - Instructions on home practice exercises | - Meditation: “The inner smile” (4) and “Total relaxation” (2), practiced alternately for 6 days - Short practice exercise: “10 Breaths for Happiness” (5), once a day - Mindfulness in one activity of daily life, once a day - Filling in a calendar of pleasant events (6) |
| 3 | Turning off the indoor radio | - Mindful movements* - Walking meditation - Meditation on thoughts* - Sharing experiences (including both session and home practice exercises) with the group - Short practice exercise: The standing mountain (7) - Sitting meditation: “The inner island”   - Sharing experiences with the group - Instructions on home practice exercises | - Meditation: “The inner smile” (4) and “Total relaxation” (2), practiced alternately for 6 days - Short practice exercise: “The standing mountain” (7), once a day - Mindfulness in an activity of daily life, once a day - Filling in calendar of pleasant events (6) - Minful movements, once a day |
| 4 | Awakening compassion | - Mindful movements* - Meditation: “Unconditional love for oneself” (8)   - Sharing experiences (including both session and home practice exercises) with the group - Self-love practice: writing a love letter to oneself - Silent meditation   - Sharing experiences with the group - Short practice exercise: Pause of the four Steps (9):   - Sharing experiences with the group - Instructions on home practice exercises | - Meditation: “Unconditional love for oneself” (8) and “Silent meditation”, practiced alternately for 6 days - Short practice exercise: “Pause of the four Steps” (9), once a day - Performing one self-care activity, daily - Minful movements, once a day |
| 5 | Taking care of my thoughts and emotions | - Mindful movements* - Sharing experiences (including both session and home practice exercises) with the group - Meditation: “Healing the pain of the past” (8) - Written exercise: How do you see this situation after this practice? - Sharing experiences with the group - Silent meditation - Sharing experiences with the group - Short practice exercise: Pause of the four Steps (9) - Instructions on home practice exercises | - Meditation: “Unconditional love for oneself” (8) and “Silent meditation”, practiced alternately for 6 days - Short practice exercise: “Pause of the four Steps” (9), once a day - Performing one self-care activity, daily - Minful movements, once a day |
| 6 | Circle of care | - Mindful movements* - Meditation: Jackal-Giraffe (10) - Sharing experiences (including both session and home practice exercises) with the group - Written exercise: end-of-program letter. What qualities have you discovered about yourself in these 6 weeks? - Sharing experiences with the group - Meditation: Metta* - Farewell. - Sharing experiences with the group - Instructions on home practice exercises |  |

*Exercises carried out with VR in the MBHC-RV group.

All course material, videos and audios are available free of charge upon request to the corresponding author.

**REFERENCES**

1. Bartley T. Mindfulness-Based Cognitive Therapy for Cancer: Gently Turning Towards. John Wiley & Sons; 2011. 438 p.

2. Hanh TN. Cómo relajarse. Editorial Kairós; 2020. 120 p.

3. Brach T. Radical Acceptance: Embracing Your Life With the Heart of a Buddha. Random House Publishing Group; 2004. 352 p.

4. Hanh TN. Las Ensenanzas del Buda: Los Tres Sutras Fundamentales. Ediciones Oniro S.A.; 2002. 288 p.

5. Schneider G. Ten Breaths to Happiness: Touching Life in its Fullness. Parallax Press; 2009. 112 p.

6. Kabat-Zinn J. Full catastrophe living: Using the wisdom of your body and mind to face stress, pain and illness. New York, NY: Delacorte; 1990.

7. Kabat-Zinn J. Wherever You Go, There You Are: Mindfulness meditation for everyday life. Hachette UK; 2016. 193 p.

8. Desmond T. Self-Compassion in Psychotherapy: Mindfulness-Based Practices for Healing and Transformation. W. W. Norton & Company; 2015. 191 p.

9. Segal ZV, Williams JMG, Teasdale JD. MBCT Terapia cognitiva basada en el mindfulness para la depresión. Editorial Kairós; 2017. 426 p.

10. Rosenberg M. Comunicación no violenta: un lenguaje de vida. Acanto; 2016. 276 p.
