# Supplementary material for: Effects of a mindfulness-based program on the occupational balance and mental health of university students. Protocol for a randomized controlled trial
Source: PLoS One. 2024 May 2;19(5):e0302018. doi: 10.1371/journal.pone.0302018 (PMC11065289; doi:10.1371/journal.pone.0302018)
Supplement: S3 Appendix — (DOCX) [file pone.0302018.s003.docx]

**Appendix 3: Research Protocol: "Effects of a Mindfulness Program on the Occupational Balance and Mental Health of University Students. Randomized Clinical Trial."** Principal Investigator (PI): Dra. Alicia Sánchez Pérez

**Introduction**

The mental health and psychological well-being of university students have become a significant public health concern (1,2). Between 29% and 37% of university students experience stress (3,4), which can lead to psychological distress with an impact on occupational balance (OB) and, in some cases, the development of psychopathologies (5).

On one hand, psychological distress, including stress, depression, and anxiety, among other manifestations, affects university populations worldwide, being associated with poor academic performance and the presence of health-risk behaviors during the university years, such as substance abuse or suicide (6). In 2016, the World Health Organization conducted a study with a sample of 5,750 university students from 21 countries and found that just over one-fifth of the participants (20.3%) met the criteria for mental disorders according to the DSM-IV/CIDI (7). Furthermore, this reality seems to have significantly worsened after the COVID-19 pandemic, as evidenced by the results of a recent meta-analysis that included 1,441,828 university students from 29 countries, showing a prevalence of depression, anxiety, and sleep disorders of 34%, 32%, and 33%, respectively (8).

On the other hand, occupational balance (OB) is defined as an individual's subjective perception of having an appropriate quantity and variety of occupations in their daily life (9). This concept is also related to overall health and well-being, life satisfaction, and low levels of stress, making it an important aspect to consider in clinical practice (10). In this regard, a study conducted with 87 undergraduate students in Argentina showed that 62% of them were dissatisfied with their daily routine (11). Similarly, a recent study with 192 university students at the University of Castilla-La Mancha reported that the students in the sample had moderate occupational balance (12).

Based on this, and considering the costly repercussions of mental health problems, psychological distress, and occupational imbalance for university students and academic institutions, an increasing number of universities currently offer some services and interventions, including mindfulness-based interventions (MBIs) (13). Mindfulness is defined as the ability to pay attention to the experience of the present moment with interest, curiosity, and acceptance (14). Mindfulness and MBIs emphasize the importance of changing our relationship with internal experiences (whether sensations, emotions, and/or thoughts) through a process of acceptance, rather than trying to eliminate or modify such experiences. It involves attention, intention, and an open and non-judgmental attitude (15), in other words, being fully aware of the present moment. It entails being connected to "here and now" and being aware of what one is feeling, thinking, and doing, which helps to appreciate each moment experienced. Through mindfulness, a change in perspective, "decentering" or "reperception" would occur, allowing the person to perceive internal experiences objectively and with great clarity (16, 17). This change in perspective facilitated by mindfulness promotes self-regulation, value clarification, cognitive, emotional, and behavioral flexibility, and the ability to face intense emotions objectively (15). In this sense, by not trying to eliminate (through mechanisms of escape or avoidance, known as "experiential avoidance") uncomfortable or unpleasant states, they paradoxically tend to dissipate more quickly, promoting calm and serenity (18,19,20).

So far, mindfulness practice has shown significant benefits in university students. A recent meta-analysis (n = 2201; 15 countries) that explored the effect of MBIs on university students showed beneficial results compared to a passive control group. Additionally, the effect size ranged from small to moderate in psychological well-being, symptoms of anxiety and depression, perceived well-being, rumination, and mindfulness skills (21).

However, the low adherence rate to MBIs among university students has been considered a significant limitation in the scientific literature (22). This fact highlights the need to identify innovative approaches, such as the incorporation of virtual reality (VR) into these interventions, to make them more appealing to this demographic (23). In this regard, a recent clinical trial that explored the effects of an MBI program in university students, including VR, showed that the group that underwent MBIs with VR had a program adherence rate of 95.7%, which is between 16.1% to 30.1% higher than the other groups that did not use VR (5).

**Rationale and project framework**

Given the low adherence of university students to MBIs, it is necessary to conduct more studies to determine whether VR can truly help MBIs overcome their typical low adherence rates. Additionally, studies are needed to enhance the monitoring of changes resulting from MBIs. Therefore, in this study where VR will be incorporated into an MBI, we also propose a mixed methodology: a quantitative part and a qualitative part. First, the quantitative approach will include, in addition to assessment through instruments adapted and validated in the Spanish context, Ecological Momentary Assessment (EMA). EMA allows the study of the relationship between individual experiences/emotions, social context, and behaviors. This "in-the-moment" and "in the current context" assessment offers several methodological advantages compared to traditional data collection systems: (1) it reduces memory bias by gathering information on current or recent experiences; (2) it enables data collection in the subject's environment, increasing compliance and reliability; (3) and it allows the detection of variations over time and factors influencing the participants' evolution. Second, the qualitative approach will explore and understand the mechanisms of change produced by the implementation of traditional MBIs and VR.

**Hypotheses**

In this study, seven hypotheses are formulated:

▪ Compared to a control group that continues with their regular routine, university students who participate in the MBI program and MBI with VR will show a significant improvement in occupational balance (Hypothesis 1: primary outcome) and a significantly greater reduction in psychological distress (anxiety, depression, and stress; Hypothesis 2: primary outcome); and these changes will be sustained at 3 months (Hypothesis 3: primary outcome).

▪ Compared to the control group, university students who undergo MBIs and MBI with VR will demonstrate improvement in variables related to mental health/illness (burden and emotional dysregulation; Hypothesis 4: secondary outcome), psychological functioning (trait mindfulness, self-compassion, life satisfaction, and acceptance; Hypothesis 5: secondary outcome), and occupations (eating habits and physical activity; Hypothesis 6: secondary outcome), and this improvement will be sustained 3 months after completing the program (Hypothesis 7: secondary outcome).

**Objectives**

The general objective of this study is to examine and compare the effects of a traditional MBI program and an MBI program with VR on the occupational balance and psychological distress of university students (i.e., stress, anxiety, and depression).

Additionally, six specific objectives are proposed:

1. To understand the participants' expectations regarding their participation in the program.
2. To examine the effects of the intervention on other variables related to mental health/illness such as burden and emotional dysregulation.
3. To examine the effects of the intervention on variables related to psychological functioning, such as trait mindfulness, self-compassion, life satisfaction, and acceptance.
4. To examine the effects of the intervention on variables related to occupation, such as eating habits and physical activity.
5. To explore whether the effects of the intervention are sustained 3 months after completing the program.
6. To understand the participants' perceptions of the program and its effects: feelings, barriers, and facilitators to attending sessions and following the program at home, satisfaction, perceived effects of participating in the program, learning outcomes, meeting expectations with the program, and suggestions for improvement.

**Methodology**

**Design**

Randomized single-blind clinical trial (RCT) with 3 arms: Traditional MBI, MBI with VR, passive control group (waitlist), with 4 assessment time points: pre-intervention assessment, inter-session, post-intervention assessment, and a 3-month follow-up.

**Participants**

The participants will be undergraduate, master's, and doctoral students at the Miguel Hernández University. The funders did not and will not have a role in study design, data collection and analysis, decision to publish, or preparation of the manuscript. They will be recruited through institutional email, informational posters, and dissemination talks.

Inclusion criteria:

1. Age 18 or older.
2. A student at the Miguel Hernández University.
3. Fluent in Spanish.
4. Signed informed consent.
5. Commitment to attending at least 66% of program sessions (4 out of 6 sessions).
6. Access to the Internet from a computer or mobile device.

Exclusion criteria:

1. Severe active mental disorder.
2. Under the influence of alcohol and other drugs during sessions and assessments.
3. Participation in another standardized meditation program during the MBI.

**Procedure**

Interested students will be interviewed by phone, and if they meet eligibility criteria, they will be informed orally and in writing (via email) about the research project. If they decide to participate and sign the informed consent, a pre-intervention assessment will be administered. Subsequently, participants will be randomly assigned to the MBI, MBI with VR, or passive control group (1:1:1 ratio) using simple randomization. Randomization will be performed by generating a random sequence with the randomizeR package in the R statistical software (24). To limit potential selection bias, research team members not involved in assessments or program implementation will generate the random sequence and inform participants by phone one week before the program begins about their assigned group. To control for possible bias, assessors will be unaware of participants' group assignments, and the MBI instructor will not know the results of the initial assessments.

**Intervention**

**Mindfulness-Based Intervention**

The mindfulness-based intervention to be implemented will be an adapted program from the Mindfulness-Based Health Care Program (MBHC). The MBHC program has a structure similar to the Mindfulness-Based Stress Reduction program (MBSR) (19) and includes some adapted practices from that program. Specifically, it includes: a) practices to cultivate attention to the somatic and sensory experience of the present moment and to cultivate a non-reactive and non-judgmental attitude toward experience; b) homework practice; and c) weekly sessions for 8 weeks. The features of MBHC that differ from MBSR include: a) shorter duration of weekly sessions, 2 hours instead of 2.5-3 hours; and b) specific practices aimed at cultivating healthy mental habits and healthy prosocial mental habits, including kindness and compassion. The MBHC program focuses on: a) paying attention to the present moment; b) cultivating acceptance and openness to the present experience without resistance and avoiding judgment; c) developing and enhancing healthy qualities such as kindness and compassion; and d) improving deeper self-inquiry by examining subjective experience through thoughts, feelings, and sensations. To provide an enriching learning environment and facilitate communication with participants, each program session includes nonviolent communication practices (25) and group dynamics based on person-centered facilitation techniques (26). All sessions will include mindful movement, formal meditation practices, informal meditation practices, sharing personal experiences and thoughts, and explanations of homework exercises. The content of each session can be found in Sánchez-Pérez et al. (27), and as an example, the material from the first session is available on the InTeO research group website (<http://inteo.edu.umh.es/atenea/ejemplo-de-sesion-de-mindfulness/>).

**Mindfulness-Based Intervention with Virtual Reality**

The program implemented will be similar to the mindfulness-based intervention, with the only difference being that one of the formal practices in each session will be conducted with VR. Additionally, participants will be provided with a device to perform VR practices at home.

**Passive Control Group**

Participants in the control group will continue to have access to psychological support services offered by the Miguel Hernández University as needed, but they will not receive any form of intervention during the research. However, for ethical reasons, once the research is completed, the passive control group will be invited to participate in an MBI free of charge.

**Variables and instruments**

This study will be conducted using mixed methodology: quantitative methodology and qualitative methodology.

**Quantitative Methodology**

Participants will complete ad hoc questionnaires, instruments adapted and validated in Spain, and will also be assessed using Ecological Momentary Assessment (EMA). Rating scales will be administered online to facilitate completion and not interfere with their daily activities.

Participants will complete assessments at the beginning (pre-intervention), during the MBI implementation (inter-session), at 6-7 weeks (post-intervention), and at 18-19 weeks (3 months of follow-up). The variables and tools used for assessment can be categorized as follows:

1. Primary Variables: a. Occupational balance. Occupational Balance Questionnaire (OBQ-E). b. Psychological distress. Depression, Anxiety, and Stress Scales (DASS-21).
2. Secondary Variables: a. Variables related to mental health/illness: i. Academic stress. Student Stress Inventory (SISCO). ii. Burnout. Maslach Burnout Inventory-Student Survey (MBI-SS). iii. Emotional dysregulation. Difficulties in Emotion Regulation Scale (DERS).

b. Variables related to psychological functioning: i. Trait mindfulness. Five Facet Mindfulness Questionnaire - Short Form (FFMQ-SF). ii. Self-compassion. Self-Compassion Scale Short Form (SCSSF). iii. Life satisfaction. Satisfaction with Life Scale (SWLS). iv. Acceptance. Acceptance and Action Questionnaire-II (AAQ-II). v. Social desirability. Marlowe-Crowne Social Desirability Scale - Short Form (DS Scale).

c. Variables related to occupations: i. Adherence to the Mediterranean Diet: Mediterranean Diet Adherence Test. ii. Physical activity. Open-ended questions included in an ad hoc questionnaire.

In addition to all the above tools, an ad hoc questionnaire with open and closed questions will be used to collect basic sociodemographic aspects of the participants, such as age, gender, highest level of education, etc.

**Ecological Momentary Assessment (EMA)**

During the MBI implementation, EMA will be conducted using a custom-designed app. The questionnaire will include 10 questions aimed at assessing momentary occupation (question 1), volition (question 2), mindfulness state (question 3), well-being state (questions 4 and 9), high and low arousal positive and negative affect (questions 5 and 6), state stress (question 7), self-compassion (question 8), recovery experience (question 10), and daily mindfulness practice monitoring (question 11).

Participants will receive a daily notification to complete the following questions. If they are unable to respond immediately, they will answer later in reference to the activity they were engaged in when they received the notification.

Questions 2 to 11 will be answered using a visual analog scale from 0 to 10 points.

1. What were you doing at this moment?
2. This activity is motivating for me.
3. I was thinking about something different from what I was doing at this moment.
4. What is my mood like right now?
5. I felt cheerful and in a good mood while doing this activity.
6. I felt calm and relaxed while doing this activity.
7. What is my stress level right now?
8. While doing the activity, I gave myself the care and kindness I needed.
9. How happy am I right now?
10. In the past 24 hours, have I engaged in any recovery activity/experience (good sleep, sports, leisure activity, etc.)?
11. In the past 24 hours, how many minutes did I dedicate to practicing mindfulness?

**Qualitative Methodology**

Ad hoc questionnaires and qualitative research through focus groups will be implemented.

In the initial questionnaires, before the intervention, qualitative questions will be included to understand the participants' expectations regarding the program. After the intervention, the focus group technique will be conducted, where semi-structured group interviews will take place. We use focus groups to gain a deeper understanding of the participants' experiences, considering that group interactions can trigger responses and generate insights that may not arise during individual interviews.

Participants will be divided into groups of 3 to 8 participants, segmented by the type of intervention received during the clinical trial and by gender, as it has been described that conducting focus groups with individuals of the same gender yields more quantity and depth in responses. Escobar and Bonilla-Jimenez recommend having a homogeneous group if the study aims to gather information from shared experiences (28).

This technique will be carried out using a flexible script of open questions by experts in the field of study, but unknown to the subjects participating, and it will be recorded using voice recording devices.

Each focus group will be conducted by two researchers (a moderator and an assistant responsible for organizational and logistical matters, and who will take written notes of events during the focus groups).

The questions will be aimed at understanding the participants' perceptions of the program and its effects: perceived feelings during its implementation, barriers and facilitators to attending the sessions and following the program at home, satisfaction, perceived effects of participating in the program, what was learned, fulfillment of expectations with the program, and suggestions for improvement.

Focus groups will continue until no new themes are identified, suggesting data saturation. Subsequently, transcription will be carried out to extract the most significant information related to the study's topic.

**Data analysis**

As this is a mixed-methods study, the data analysis procedures for the quantitative and qualitative parts of the study are described separately.

**Quantitative Data Analysis**

Statistical analysis will be performed using the R software, version 4.1.1 (R Foundation for Statistical Computing, Vienna, Austria; [http://www.R-project.org](http://www.r-project.org/)). All statistical tests will be two-tailed with a significance level set at 0.05. All data analyses will be conducted using an "intent-to-treat" approach to ensure the initial comparability between groups obtained through randomization, thus reducing potential biases.

General characteristics of study participants will be described as frequencies and percentages (categorical variables) and as mean and standard deviation when the distribution is normal, or median and interquartile range when it is not (quantitative variables). The distribution of quantitative variables will be assessed using the Kolmogorov-Smirnov test corrected by Lilliefors.

To explore differences between intervention and control groups regarding primary and secondary variables, we will use the chi-square test or Fisher's exact test for categorical variables and Student's t-test or Mann-Whitney U test for continuous variables. To control for confounding bias, bivariate regression models will be used to assess the effect on primary outcomes between study groups using all covariates that are significant (p < 0.20) to build core models. Additionally, following a backward elimination procedure, all variables associated with primary outcomes at a level of p < 0.10 will be included. The aforementioned variables, even if not statistically significant, will be kept in the models if they change the magnitude of the primary effects by more than 10%. Finally, to assess the effect of the MBHC intervention on outcomes at baseline, post-intervention, and follow-up, multiple regression models will be estimated.

**Qualitative Data Analysis**

Data analysis will be conducted following the modified grounded theory approach (29).

Interviews will be transcribed verbatim, and their accuracy will be verified. They will be imported into the MAXQDA program, where the analysis will be performed. A thematic index (coding) will be constructed and applied independently to the initial transcriptions by three researchers.

The following process will be followed:

1. Selection of the most significant phrases.
2. Initial grouping of significant phrases into categories and themes.
3. Creation of identifying codes for categories and themes.
4. Validation of categories and redefinition if necessary.

Subsequently, the three researchers will again check the appropriateness of data interpretation to ensure systematic and verifiable data allocation.

Finally, a conceptual map will be created, and the data will be interpreted, explaining the patterns of association.

**Ethical considerations**

This study will be conducted following recognized guidelines in the Declaration of Helsinki (52nd General Assembly Edinburgh, Scotland, October 2000), Good Clinical Practice Guidelines, and in compliance with current Spanish legal regulations governing clinical research in humans (Royal Decree 561/1993), as well as Organic Law 3/2018, of December 5, 2018, on the Protection of Personal Data and guarantee of digital rights (<https://www.boe.es/eli/es/lo/2018/12/05/3>). Additionally, this study will be registered on ClinicalTrials.gov (<https://clinicaltrials.gov/>) and has already the approval from the Research Ethics and Integrity Committee of the Miguel Hernández University.

All participants will be verbally and in writing informed about the study, and their participation will be entirely voluntary. They will all provide informed consent and will not receive any incentives for participating in this study.

The confidentiality of the information collected will be guaranteed throughout the research process (data collection, analysis, and dissemination of results, among others). Information on participants' personal data will be incorporated and processed in a computerized database, complying with the guarantees established by current legislation. Additionally, the information to be analyzed will be dissociated, from the outset, from participants' personal data, so each participant will be assigned an identifying code. All information will be appropriately safeguarded by the study's principal investigator.

**Study limitations**

An important limitation is that students randomly assigned to the intervention group will receive an informational talk on mindfulness, particularly on the MBHC program, before the study begins. After this talk, students will decide whether or not they want to continue in the study. Although this may imply a potential selection bias by compromising the randomization process of the study, we hope to minimize this inconvenience by adequately informing potential participants about the project through the informed consent form and the information sheet during the recruitment period. As for the control group, they are likely to engage in social, leisure, and/or recreational activities, including yoga, relaxation techniques, etc., which can easily introduce intervention biases. However, we will collect information about activities regularly practiced during leisure time to control for the potential influence of these variables.

Lastly, although a probable dropout rate is used in the design of this clinical trial, we are aware that dropouts may occur during the study, which compromises the validity of the results. Another strategy to reduce the dropout rate that can be adopted is that participants who do not attend the post-intervention assessment will be called again for the follow-up assessment to avoid data loss. In the event of missing data, multiple imputations will be implemented using chained equations.

**Schedule**

The study will be conducted over the three years following the approval by the Ethics Committee.

During the first year, tasks will be planned, including the initiation of the feasibility study and the pilot study, as well as meetings of the research team for sharing, discussion, and project monitoring.

During the second year, participants for the research study will be recruited, randomized into different groups, and the corresponding mindfulness-based interventions will be implemented. Additionally, the quantitative and qualitative evaluation process will take place, along with team meetings for sharing, discussion, and result monitoring.

During the third year, data cleansing of the resulting database will be carried out, followed by the corresponding analysis to address the proposed study objectives. In addition, various communications with the study results will be prepared and presented at different scientific forums (conferences, workshops, outreach events, etc.), both scientific and non-scientific. Furthermore, the writing of two manuscripts with the study results and their subsequent publication in high-impact factor journals in the JCR (Journal Citation Reports) will take place. Similar to the previous years, team meetings will be held for sharing, discussion, and result monitoring.

**REFERENCES**

1. Sheldon E, Simmonds-Buckley M, Bone C, Mascarenhas T, Chan N, Wincott M, et al. Prevalence and risk factors for mental health problems in university undergraduate students: A systematic review with meta-analysis. J Affect Disord. 2021;287:282-92.

2. Brown P. The invisible problem? Improving students’ mental health. :66.

3. Yusoff MSB, Abdul Rahim AF, Yaacob MJ. Prevalence and Sources of Stress among University Sains Malaysia Medical Students. Malays J Med Sci MJMS. 2010;17(1):30-7.

4. Yf J, Yt L. PREVALENCE AND DETERMINANTS OF PERCEIVED STRESS AMONG UNDERGRADUATE STUDENTS IN A MALAYSIAN UNIVERSITY. J Health Transl Med [Internet]. 2018 [citado 7 de diciembre de 2022];21(1). Disponible en: https://jummec.um.edu.my/index.php/jummec/article/view/11016

5. Modrego-Alarcón M, López-Del-Hoyo Y, García-Campayo J, Pérez-Aranda A, Navarro-

Gil M, Beltrán-Ruiz M, et al. Efficacy of a mindfulness-based programme with and

without virtual reality support to reduce stress in university students: A randomized

controlled trial. Behav Res Ther. 2021; 142:103866.

6. Sharp J, Theiler S. A Review of Psychological Distress Among University Students:

Pervasiveness, Implications and Potential Points of Intervention. Int J Adv Couns.

2018;40(3):193-212.

7. Auerbach RP, Alonso J, Axinn WG, Cuijpers P, Ebert DD, Green JG, et al. Mental

disorders among college students in the World Health Organization World Mental

Health Surveys. Psychol Med. 2016;46(14):2955-70.

8. Deng J, Zhou F, Hou W, Silver Z, Wong CY, Chang O, et al. The prevalence of depressive

symptoms, anxiety symptoms and sleep disturbance in higher education students

during the COVID-19 pandemic: A systematic review and meta-analysis. Psychiatry

Res. 2021; 301:113863.

9. Gómez PP. Equilibrio ocupacional en estudiantes de terapia ocupacional [Internet]

[http://purl.org/dc/dcmitype/Text]. Universidad Miguel Hernández; 2017 [citado 3

de marzo de 2021]. Disponible en:

https://dialnet.unirioja.es/servlet/tesis?codigo=109869

10.Dhas BN, Wagman P. Occupational balance from a clinical perspective. Scand J Occup

Ther. 2022;29(5):373-9.

11.Demiryi M, Berezin S, Fernández G, Ganso H, Gaiteiro A. Primera etapa del proyecto

de investigación. Las rutinas diarias y el balance ocupacional de los alumnos de la

Universidad Nacional de Quilmes.. Rev Chil Ter Ocupacional.

2013;13(2):.g. 73-78.

12.Romero-T.bar A, Rodríguez-Hernández M, Segura-Fragoso A, Cantero-Garlito PA.

Analysis of Occupational Balance and Its Relation to Problematic Internet Use in

University Occupational Therapy Students. Healthc Basel Switz.

2021;9(2):197.

13.Regehr C, Glancy D, Pitts A. Interventions to reduce stress in university students: a

review and meta-analysis. J Affect Disord. 2013;148(1):1-11.

14.Levit-Binnun N, Arbel K, Dorjee D. The Mindfulness Map: A Practical Classification

Framework of Mindfulness Practices, Associated Intentions, and Experiential

Understandings. Front Psychol. 2021;12:727857.

15.Shapiro SL, Carlson LE, Astin JA, Freedman B. Mechanisms of mindfulness. J Clin

Psychol. 2006;62(3):373-86.

16.Baer RA. Mindfulness training as a clinical intervention: A conceptual and empirical

review. Clin Psychol Sci Pract. 2003;10:125-43.

17.Brown KW, Ryan RM, Creswell JD. Mindfulness: Theoretical foundations and

evidence for its salutary effects. Psychol Inq. 2007;18:211-37.

18.Bishop SR. What Do We Really Know About Mindfulness-Based Stress Reduction?

Psychosom Med. 2002;64(1):71-83.

19.Kabat-Zinn J. Full catastrophe living: Using the wisdom of your body and mind to face

stress, pain and illness. New York, NY: Delacorte; 1990.

20.Kabat-Zinn J. Wherever You Go, There You Are: Mindfulness Meditation in Everyday

Life. Hachette Books; 1994. 132 p.

21.Dawson AF, Brown WW, Anderson J, Datta B, Donald JN, Hong K, et al. Mindfulness-

Based Interventions for University Students: A Systematic Review and Meta-Analysis

of Randomised Controlled Trials. Appl Psychol Health Well-Being.

2020;12(2):384-410.

22.Pedrelli P, Nyer M, Yeung A, Zulauf C, Wilens T. College Students: Mental Health

Problems and Treatment Considerations. Acad Psychiatry J Am Assoc Dir Psychiatr

Resid Train Assoc Acad Psychiatry. 2015;39(5):503-11.

23.Furlong J, Davies C. Young people, new technologies and learning at home: Taking

context seriously. Oxf Rev Educ. 2012;38:45-62.

24.Uschner D, Schindler D, Hilgers RD, Heussen N. randomizeR: An R Package for the

Assessment and Implementation of Randomization in Clinical Trials. J Stat Softw.

2018;85(8):1-22.

25.Rosenberg M. Comunicación no violenta: un lenguaje de vida. Acanto; 2016. 276 p.

26. Rogers CR. Grupos de encuentro. Amorrortu; 2018. 192 p.16

27.S.nchez-P.rez A, Mendialdua-Canales D, Hurtado-Pomares M, Peral-Gómez P,

Juárez-Leal I, Espinosa-Sempere C, et al. The ATENción Plena en Enfermedad de

Alzheimer (ATENEA-Mindfulness in Alzheimer’s Disease) Program for Caregivers:

Study Protocol for a Randomized Controlled Trial. Healthc Basel Switz.2022;10(3):542.

28.Bonilla-Jimenez FI, Escobar J. Grupos focales: una guía conceptual y metodológica. 2017 [citado 7 de diciembre de 2022]; Disponible en:

http://148.202.167.116:8080/xmlui/handle/123456789/957

29.Corbin J, Strauss A. Basics of Qualitative Research: Techniques and Procedures for

Developing Grounded Theory. SAGE Publications; 2014. 457 p.

Principio del formulario

Final del formulario
