# Supplementary material for: Effects of a mindfulness-based program on the occupational balance and mental health of university students. Protocol for a randomized controlled trial
Source: PLoS One. 2024 May 2;19(5):e0302018. doi: 10.1371/journal.pone.0302018 (PMC11065289; doi:10.1371/journal.pone.0302018)
Supplement: S4 Appendix — (DOCX) [file pone.0302018.s004.docx]

**Appendix 4. Protocolo de investigación: “Efectos de un programa de mindfulness en el equilibrio ocupacional y la salud mental de los estudiantes universitarios. Ensayo clínico aleatorizado.”**

**Investigador principal (IP):** **Dra. Alicia Sánchez Pérez**

**Introducción**

La salud mental y el bienestar psicológico de las personas que cursan estudios universitarios se ha convertido en una importante preocupación para la salud pública [(1](https://www.zotero.org/google-docs/?ihAhH5),[2)](https://www.zotero.org/google-docs/?sdwASz). Entre un 29% y un 37% de los estudiantes universitarios presentan estrés [(3](https://www.zotero.org/google-docs/?b4FJoU),[4)](https://www.zotero.org/google-docs/?DhyeKx), lo cual puede inducir hacia un malestar psicológico con impacto en el equilibrio ocupacional (EO) y, en algunos casos, hacia el desarrollo de psicopatologías [(5)](https://www.zotero.org/google-docs/?EJDMid).

Por un lado, el malestar psicológico, incluyendo estrés, depresión y ansiedad entre otras manifestaciones, afecta a la población universitaria de todo el mundo, relacionándose con el bajo rendimiento académico y la presencia de comportamientos de riesgo para la salud durante la etapa universitaria, como el consumo de sustancias o el suicidio [(6)](https://www.zotero.org/google-docs/?nMOheh). En 2016, la Organización Mundial de la Salud realizó un estudio con una muestra de 5750 estudiantes universitarios procedentes de 21 países y concluyó que algo más de una quinta parte de las personas participantes (20,3%) cumplía los criterios de trastorno mental según el DSM-IV/CIDI [(7)](https://www.zotero.org/google-docs/?YAiyxv). Asimismo, esta realidad parece haber empeorado notablemente tras la pandemia de la COVID-19 según los resultados de un metaanálisis reciente que incluía a 1.441.828 estudiantes universitarios de 29 países, y cuyos resultados mostraron una prevalencia de depresión, ansiedad y trastornos del sueño del 34%, 32% y 33%, respectivamente [(8)](https://www.zotero.org/google-docs/?NkD0sf).

Por otro lado, el equilibrio ocupacional (EO) se define como la percepción subjetiva de un individuo de tener una adecuada cantidad y variedad de ocupaciones en su vida diaria [(9)](https://www.zotero.org/google-docs/?OHOchS). Este concepto está relacionado, además, con la salud y el bienestar general, con la satisfacción con la vida y con bajos niveles de estrés, lo que lo convierte en un aspecto importante a considerar en la práctica clínica [(10)](https://www.zotero.org/google-docs/?naeIqp). En este sentido, un estudio realizado con 87 estudiantes de pregrado en Argentina mostró que el 62% de ellos se sentía insatisfecho con su rutina diaria [(11)](https://www.zotero.org/google-docs/?iT3v4p). En la misma línea, un estudio reciente llevado a cabo con 192 universitarios de la Universidad de Castilla-La Mancha informó que los estudiantes de la muestra presentaban un equilibrio ocupacional moderado [(12)](https://www.zotero.org/google-docs/?bDyOHp).

En base a ello, y teniendo en cuenta las costosas repercusiones de los problemas de salud mental, malestar psicológico y desequilibrio ocupacional para las personas que cursan estudios universitarios y para las instituciones académicas, un número creciente de universidades ofrece en la actualidad algunos servicios e intervenciones, entre las que se incluyen las intervenciones basadas en la atención plena o mindfulness (MBIs) [(9](https://www.zotero.org/google-docs/?T0iTQ9)). La atención plena o mindfulness se define como la capacidad de prestar atención a la experiencia del momento presente con interés, curiosidad y aceptación [(14)](https://www.zotero.org/google-docs/?RPFMft) y, hasta el momento, su práctica ha mostrado importantes beneficios en estudiantes universitarios. Un reciente metaanálisis (n = 2201; 15 países) que exploró el efecto de MBIs en estudiantes universitarios mostró resultados beneficiosos de las mismas al comparar sus efectos con un grupo control pasivo. Asimismo, el tamaño del efecto fue de pequeño a moderado en bienestar psicológico, síntomas de ansiedad y depresión, bienestar percibido, rumiación y habilidades de atención plena [(15)](https://www.zotero.org/google-docs/?jFd8xC).

No obstante, el bajo nivel de adherencia a las MBIs del estudiantado universitarios ha sido considerado en la literatura científica como una importante limitación [(16)](https://www.zotero.org/google-docs/?AULxWp). Este hecho pone de manifiesto la necesidad de identificar formas innovadoras, tales como la incorporación de la realidad virtual (RV) a estas intervenciones, que permitan hacerlas más atractivas para este colectivo [(17)](https://www.zotero.org/google-docs/?rFiOgm). En este sentido, un ensayo clínico reciente que exploraba los efectos de un programa MBIs en estudiantes universitarios incluyendo la RV mostró que el grupo que realizó MBIs con RV presentó una tasa de adherencia al programa del 95,7%; es decir, entre un 16,1% - 30,1% mayor al resto de grupos que no utilizó RV [(5)](https://www.zotero.org/google-docs/?GyPao4).

**Justificación y marco del proyecto**

Dada la baja adherencia de los estudiantes universitarios a los MBIs, resulta necesario realizar más estudios que permitan comprobar si la RV puede realmente ayudar a los MBIs a superar sus típicas bajas tasas de adhesión. Asimismo, son necesarios estudios que permitan mejorar la monitorización de los cambios producidos por los MBIs.

Por ello, en este estudio en el que se incorporará RV a una MBI, nos planteamos además una metodología mixta: una parte cuantitativa y una parte cualitativa. En primer lugar, el abordaje cuantitativo incluirá, además de la evaluación a través de instrumentos de evaluación adaptados y validados en el contexto español, la Evaluación Ecológica Momentánea (EMA). La EMA permite estudiar la relación entre las experiencias/emociones individuales, el contexto social y los comportamientos. Esta evaluación “en el momento” y “en el contexto actual” presenta múltiples ventajas metodológicas comparada con sistemas tradicionales de recogida de datos: (1) reduce el sesgo de memoria obteniendo información de experiencia actuales o recientes; (2) permite la recogida de información en el medio del sujeto, incrementando la cumplimentación y fiabilidad; (3) y permite la detección de variaciones a lo largo del tiempo y de factores que influyan en la evolución de las personas participantes. En segundo lugar, el abordaje cualitativo permitirá explorar y comprender los mecanismos de cambio producidos con la implementación de los MBIs tradicionales y con RV.

**Hipótesis**

En este estudio se formulan siete hipótesis:

- En comparación con un grupo de control que continuará con su rutina habitual, los estudiantes universitarios que realicen el programa MBI y MBI con RV mostrarán una mejora significativa en el equilibrio ocupacional (Hipótesis 1: resultado primario) y una disminución significativamente mayor del malestar psicológico (ansiedad, depresión y estrés; Hipótesis 2: resultado primario); y estos cambios se mantendrán a los 3 meses (Hipótesis 3: resultado primario).
- En comparación con el grupo control, los estudiantes universitarios que realicen el MBIs y MBI con RV, mostrarán una mejora en variables relacionadas con la salud/enfermedad mental (sobrecarga y desregulación emocional; Hipótesis 4: resultado secundario), con el funcionamiento psicológico (atención plena rasgo, autocompasión, satisfacción con la vida y aceptación; Hipótesis 5: resultado secundario) y con las ocupaciones (hábitos de alimentación y actividad física; Hipótesis 6: resultado secundario), y esta mejora se mantendrá a los 3 meses tras finalizar el programa (Hipótesis 7: resultado secundario).

**Objetivos**

El objetivo general del presente estudio será examinar y comparar los efectos de un programa de MBIs tradicional y de un programa un MBIs con RV sobre el equilibrio ocupacional y el malestar psicológico de los estudiantes universitarios (es decir el estrés, la ansiedad y la depresión).

Asimismo, se proponen seis objetivos específicos:

1. Conocer las expectativas de los participantes con respecto a su participación en el programa.
2. Examinar los efectos de la intervención en otras variables relacionadas con la salud/enfermedad mental como la sobrecarga y la desregulación emocional.
3. Examinar los efectos de la intervención en variables relacionadas con el funcionamiento psicológico como la atención plena-rasgo, la autocompasión, la satisfacción con la vida y la aceptación.
4. Examinar los efectos de la intervención en variables relacionadas con la ocupación, como los hábitos de alimentación y la actividad física.
5. Explorar si los efectos de la intervención se mantienen a los 3 meses tras finalizar el programa.
6. Conocer las percepciones de los participantes sobre el programa y sus efectos: sentimientos, barreras y facilitadores para asistir a las sesiones y para seguir el programa en casa, satisfacción, efectos percibidos con la realización del programa, aprendizajes, cumplimiento de expectativas con el programa y sugerencias de mejora.

**Metodología**

**Diseño**

Ensayo clínico aleatorizado (ECA) simple ciego de 3 brazos: MBI tradicional, MBI con RV, grupo control pasivo (lista de espera), con 4 momentos de evaluación: evaluación pre intervención, inter-sesión, post intervención y seguimiento a los 3 meses.

**Participantes**

Los participantes serán estudiantes de grado, máster y doctorado de la Universidad Miguel Hernández de Elche. Todos ellos serán reclutados a través del correo institucional, carteles informativos y charlas de difusión.

Los criterios de inclusión serán:

1. Ser mayor de 18 años.
2. Ser estudiante de la Universidad Miguel Hernández de Elche.
3. Hablar castellano de forma fluida.
4. Firmar el consentimiento informado.
5. Compromiso de asistencia a al menos un 66% de las sesiones del programa (4 de las 6 sesiones).
6. Acceso a Internet desde un ordenador o dispositivo móvil.

Los criterios de exclusión serán:

1. Trastorno mental grave en fase activa.
2. Estar bajo la influencia del alcohol y otras drogas durante las sesiones y evaluaciones.
3. Participación en otro programa estandarizado de meditación durante la MBI.

**Procedimiento**

Los estudiantes interesados serán entrevistados telefónicamente y, si cumplen los criterios de elegibilidad, se les informará de forma oral y por escrito (vía email) sobre el proyecto de investigación. Si deciden participar, una vez firmen el consentimiento informado, se les administrará la evaluación pre-intervención.

Posteriormente, los participantes serán asignados mediante aleatorización simple al grupo de intervención MBI, MBI con RV o al grupo de control pasivo (proporción 1:1:1). La aleatorización se realizará generando una secuencia aleatoria con el paquete *randomizeR* del software estadístico R [(18)](https://www.zotero.org/google-docs/?JqNK6O).

Para limitar el potencial sesgo de selección, los miembros del equipo de investigación que no estén involucrados en las evaluaciones y la implementación del programa de intervención se encargarán de generar la secuencia aleatoria e informarán a los participantes por teléfono una semana antes del inicio del programa sobre el grupo al que han sido asignados. Para controlar el posible sesgo, los evaluadores desconocerán la asignación de los participantes en el estudio, y el instructor de los MBIs no conocerá los resultados de las evaluaciones iniciales.

**Intervención**

**Intervención Basada en Mindfulness**

La intervención basada en mindfulness que se implementará será un programa adaptado del Programa de Cuidado de la Salud Basado en Mindfulness (MBHC).

El programa MBCH tiene una estructura similar a la del programa de reducción del estrés basado en la atención plena (MBSR) [(19)](https://www.zotero.org/google-docs/?LfDPcx), e incluye algunas prácticas adaptadas de ese programa. En concreto, incluye: a) prácticas para cultivar la atención a la experiencia somática y sensorial del momento presente y para cultivar una actitud no reactiva y sin juicios hacia la experiencia; b) práctica en casa; y c) sesiones una vez a la semana durante 8 semanas. Las características de la MBCH que difieren de la MBSR incluyen: a) la duración de las sesiones semanales es más corta, 2 horas, en lugar de 2,5-3 horas; y b) prácticas específicas destinadas a cultivar hábitos mentales saludables y hábitos mentales prosociales saludables, incluyendo la amabilidad y la compasión.

El programa MBHC se centra en: a) prestar atención al momento presente; b) cultivar la aceptación y la apertura a la experiencia presente sin resistencia y evitando el juicio; c) desarrollar y potenciar cualidades saludables como la amabilidad y la compasión; y d) mejorar la autoindagación más profunda examinando la experiencia subjetiva a través de pensamientos, sentimientos y sensaciones. Para proporcionar un entorno de aprendizaje enriquecedor y facilitar la comunicación con los participantes, en cada sesión del programa se implementan prácticas de comunicación no violenta [(20)](https://www.zotero.org/google-docs/?jXgqWQ) y dinámicas de grupo basadas en técnicas de facilitación centradas en la persona [(21)](https://www.zotero.org/google-docs/?tRxyVN). Todas las sesiones incluirán movimiento con atención plena, prácticas formales de meditación, prácticas informales de meditación, puesta en común de experiencias y pensamientos personales, y explicación de ejercicios en casa. El contenido de cada una de las sesiones puede consultarse en [(22)](https://www.zotero.org/google-docs/?UqC71a) y, como ejemplo, el material de la primera sesión está disponible en la página web del grupo de investigación InTeO (<http://inteo.edu.umh.es/atenea/ejemplo-de-sesion-de-mindfulness/>).

**Intervención Basada en Mindfulness con realidad virtual**

El programa implementado será similar a la intervención basada en mindfulness, con la única diferencia de que una de las prácticas formales de cada sesión se realizará con RV. Además, se facilitará a los participantes un dispositivo para poder realizar las prácticas en casa con RV.

**Grupo control pasivo**

Los participantes del grupo control continuarán accediendo cuando lo precisen a los servicios de atención psicológica ofrecidos por la Universidad Miguel Hernández de Elche, pero no recibirán ningún tipo de intervención durante la investigación. No obstante, por razones éticas, una vez finalizada la investigación, se invitará al grupo de control pasivo a participar en un MBI de forma gratuita.

**Variables e instrumentos**

Este estudio se realizará con metodología mixta: metodología cuantitativa y metodología cualitativa.

**Metodología cuantitativa**

Se administrarán a los participantes cuestionarios *ad hoc*, instrumentos de medida adaptados y validados en España, y se les evaluará adicionalmente mediante Evaluación Ecológica Momentánea (EMA). Las escalas de valoración se administrarán en formato on-line para facilitar su cumplimentación y no interferir en sus ocupaciones diarias.

Los participantes completarán una evaluación al inicio (pre intervención), durante la implementación del MBI (inter-sesión), a las 6-7 semanas (post-intervención) y a las 18-19 semanas (3 meses de seguimiento). Las variables y herramientas utilizadas para su evaluación puede clasificarse de la siguiente manera:

1. Variables principales:
   1. Equilibrio ocupacional. Cuestionario de Equilibrio Ocupacional (OBQ-E).
   2. Malestar psicológico. Escalas de Depresión, Ansiedad y Estrés (DASS-21).
2. Variables secundarias:
   1. Variables relacionadas con la salud/enfermedad mental:
      1. Estrés académico. Inventario SISCO de Estrés Académico.
      2. Sobrecarga. Cuestionario Maslach Burnout Inventory-Student Survey (MBI-SS).
      3. Desregulación emocional. Escala de dificultades en regulación emocional (DERS).
   2. Variables relacionadas con el funcionamiento psicológico:
      1. Atención plena-rasgo. Cuestionario de las Cinco Facetas del Mindfulness - Versión Reducida (FFMQ-SF).
      2. Autocompasión. Escala de Autocompasión versión reducida (SCS-SF).
      3. Satisfacción con la vida. Escala de Satisfacción con la Vida (SWLS).
      4. Aceptación. Cuestionario de Aceptación y Acción-II (AAQ-II).
      5. Deseabilidad social. Escala de Deseabilidad Social de Marlowe-Crowne versión reducida. (Escala DS).
   3. Variables relacionadas con las ocupaciones:
      1. Adherencia a la Dieta Mediterránea: Test de Adherencia a la dieta mediterránea.
      2. Actividad física. Preguntas abiertas incluidas en un cuestionario *ad hoc*.

Además de todas las herramientas anteriores se utilizará un cuestionario *ad hoc* con preguntas abiertas y cerradas para recoger los aspectos sociodemográficos básicos de las personas participantes, tales como la edad, el sexo, el nivel máximo de estudios, etc.

Evaluación Ecológica Momentánea (EMA)

Durante la implementación del MBI, se evaluará mediante EMA con una app diseñada ad hoc. El cuestionario incluirá 10 preguntas con el objetivo de evaluar de forma momentánea la ocupación (pregunta 1), volición (pregunta 2), mindfulness estado (pregunta 3), estado de bienestar (pregunta 4 y 9), afecto positivo y negativo de alto y bajo arousal (preguntas 5 y 6), estrés estado (pregunta 7), autocompasión (pregunta 8), experiencia de recuperación (pregunta 10) y monitorización de la práctica de mindfulness diaria (pregunta 11).

Los participantes recibirán un aviso diario para cumplimentar las siguientes preguntas. En el caso de no poder responder de forma inmediata, contestarán posteriormente en referencia a la actividad que estaban llevando a cabo en el momento que les llegó el aviso.

Las preguntas de la 2 a la 11 se responderán mediante una escala analógica visual de 0 a 10 puntos.

1. ¿Qué estaba haciendo en este momento?
2. Esta actividad es motivadora para mí
3. Estaba pensando en algo diferente a lo que estaba haciendo en este momento
4. ¿Cuál es mi estado de ánimo en este momento?
5. Me he sentido alegre y de buen humor mientras hacía esa actividad
6. Me he sentido tranquilo y relajado mientras hacía esa actividad
7. ¿Cuál es mi nivel de estrés en este momento?
8. Mientras hacía la actividad me he dado el cuidado y la amabilidad que necesito
9. ¿Cuál es mi nivel de felicidad en este momento?
10. En las últimas 24 horas he realizado alguna actividad/experiencia de recuperación (he dormido bien, he hecho deporte, actividad de ocio, etc)
11. En las últimas 24 horas, ¿cuántos minutos he dedicado a practicar mindfulness?

**Metodología cualitativa**

Se implementarán cuestionarios *ad hoc* e investigación cualitativa mediante grupos focales.

En los cuestionarios iniciales, previos a la intervención, se incluirán preguntas cualitativas para conocer las expectativas de los participantes con el programa.

Posterior a la intervención se realizará la técnica de grupos focales, donde se llevarán a cabo entrevistas grupales semiestructuradas. Utilizamos grupos focales para obtener una comprensión más profunda de las experiencias de los participantes, considerando que las interacciones grupales pueden desencadenar respuestas y generar conocimientos que pueden no surgir durante las entrevistas individuales.

Los participantes se distribuirán en grupos de entre 3 y 8 participantes, segmentados por tipo de intervención recibida durante el ensayo clínico y por sexo, ya que se ha descrito que la realización de grupos focales con personas del mismo sexo genera mayor cantidad y profundidad en las respuestas. Escobar y Bonilla-Jimenez recomiendan tener un grupo homogéneo si el estudio tiene como objetivo recabar información que provenga de experiencias compartidas [(23)](https://www.zotero.org/google-docs/?JHBJPE).

Esta técnica será llevada a cabo mediante un guión flexible de preguntas abiertas por expertos en el tema de estudio, pero desconocidas por los sujetos que participan, y se registrará mediante dispositivos de grabación de voz.

Cada grupo focal será realizado por dos investigadores (un moderador y un asistente que se encargará de los temas organizativos y logísticos, y tomará notas escritas de aspectos acontecidos durante los grupos focales).

Las preguntas irán dirigidas a conocer las percepciones de los participantes sobre el programa y sus efectos: sentimientos percibidos durante su realización, barreras y facilitadores para asistir a las sesiones y para seguir el programa en casa, satisfacción, efectos percibidos con la realización del programa, aprendizajes, cumplimiento de expectativas con el programa y sugerencias de mejora.

Se continuará la realización de grupos focales hasta que no se identifiquen nuevos temas, lo que sugerirá saturación de datos. Posteriormente, se procederá a su transcripción para poder extraer la información más significativa con el tema de estudio.

**Análisis de los datos**

Al tratarse de un estudio con metodología mixta, a continuación se describe el procedimiento de análisis de datos del estudio cuantitativo y cualitativo.

**Análisis de datos cuantitativos**

El análisis estadístico se realizará con el programa informático R, versión 4.1.1 (R Foundation for Statistical Computing, Viena, Austria; [http://www.R-project.org](http://www.r-project.org)). Todas las pruebas estadísticas serán bilaterales con un nivel de significación fijado en 0,05. Todos los análisis de datos se llevarán a cabo utilizando un enfoque de "intención de tratar" para garantizar la comparabilidad inicial entre grupos obtenida mediante aleatorización, reduciendo así los posibles sesgos.

Las características generales de los participantes en el estudio se describirán como frecuencias y porcentajes (variables categóricas) y como media y desviación estándar, cuando la distribución sea normal, o mediana y rango intercuartílico, cuando no lo sea (variables cuantitativas). La distribución de las variables cuantitativas se evaluará mediante la prueba de Kolmogorov-Smirnov corregida por Lilliefors.

Para explorar las diferencias entre los grupos de intervención y control con respecto a las variables principales y secundarias, utilizaremos la prueba de Chi-cuadrado o la prueba exacta de Fisher para las variables categóricas, y la prueba t de Student o la prueba U de Mann Whitney para las variables continuas. Para controlar el sesgo de confusión, se utilizarán modelos de regresión bivariante para evaluar el efecto sobre los resultados primarios entre los grupos de estudio utilizando todas las covariables significativas (p <0,20) para construir los modelos centrales. Además, siguiendo un procedimiento de eliminación hacia atrás, se incluirán todas las covariables asociadas con los resultados principales a un nivel de p <0,10. Las variables anteriores, aunque no sean estadísticamente significativas, se mantendrán en los modelos si modificaron la magnitud de los efectos principales en más de un 10%. Por último, para evaluar el efecto de la intervención MBHC sobre los resultados al inicio, post-intervención y en el seguimiento, se estimarán modelos de regresión múltiple.

**Análisis de datos cualitativos**

El análisis de los datos se llevará a cabo siguiendo el enfoque de la teoría fundamentada modificada [(24)](https://www.zotero.org/google-docs/?vNYcmE).

Las entrevistas se transcribirán textualmente y se verificará su exactitud. Se importarán al programa MAXQDA, desde el que se realizará el análisis. Se construirá un índice temático (codificación), y se aplicará de forma independiente a las primeras transcripciones por parte de tres investigadores.

Se seguirá el siguiente proceso:

1. Selección de las frases más significativas.
2. Agrupación inicial de las frases significativas en categorías y temas.
3. Creación de códigos identificativos de las categorías y temas.
4. Validación de las categorías y redefinición, en su caso.

Posteriormente, los tres investigadores de nuevo comprobarán la adecuación de la

interpretación de los datos para asegurar que la asignación de los datos sea sistemática y verificable.

Finalmente, se realizará un mapa conceptual y se interpretarán los datos explicando los patrones de asociación.

**Aspectos éticos**

Este estudio se llevará a cabo siguiendo las normas reconocidas en la Declaración de Helsinki (52ª Asamblea General Edimburgo, Escocia, Octubre 2000), las Normas de Buena Práctica Clínica y en cumplimiento de la normativa legal vigente española que regula la investigación clínica en humanos (Real Decreto 561/1993), así como de la Ley Orgánica 3/2018, de 5 de diciembre, de Protección de Datos Personales y garantía de los derechos digitales (<https://www.boe.es/buscar/act.php?id=BOE-A-2018-16673>) vigente en España. Asimismo, este estudio será registrado en ClinicalTrials.gov (<https://clinicaltrials.gov/>) y ya ha recibido la aprobación del Comité de Ética e Integridad en la Investigación de la Universidad Miguel Hernández.

Todas las personas participantes serán informadas verbalmente y por escrito sobre el estudio y su participación será totalmente voluntaria. Todos/as ellos/as proporcionarán un consentimiento informado y no obtendrán ningún incentivo por participar en este estudio.

La confidencialidad de la información recogida se garantizará durante todo el proceso de investigación (recopilación de datos, análisis de estos y/o difusión de los resultados, entre otros). La información sobre los datos personales de los/as participantes será incorporada y tratada en una base de datos informatizada cumpliendo con las garantías que establece la legislación vigente. Adicionalmente, la información a analizar será disociada, desde el primer momento, de los datos personales de los/as participantes, por lo que a cada participante se le asignará un código identificador. Toda la información permanecerá debidamente custodiada por la IP del estudio.

**Limitaciones de estudio**

Una limitación importante es que los estudiantes asignados aleatoriamente al grupo de intervención recibirán una charla informativa sobre mindfulness, en particular sobre el programa MBHC, antes de comenzar el estudio. Tras esta charla, los estudiantes decidirán si desean o no continuar en el estudio. Aunque esto puede implicar un potencial sesgo de selección al comprometer el proceso de aleatorización del estudio, esperamos minimizar este inconveniente informando adecuadamente a los potenciales participantes sobre el proyecto a través del formulario de consentimiento informado y la hoja informativa durante el periodo de reclutamiento. En cuanto al grupo control, es probable que realicen actividades sociales, de ocio y/o recreativas, incluyendo yoga, técnicas de relajación, etc., lo que hace que fácilmente puedan producirse sesgos de intervención. Sin embargo, recogeremos información sobre las actividades practicadas regularmente en el tiempo libre para controlar la probable influencia de estas variables. Por último, aunque una probable tasa de abandono se utilice en el cálculo en el diseño de este ensayo clínico, somos conscientes de que los abandonos pueden ocurrir durante el estudio, lo que compromete la validez de los resultados. Otra estrategia para reducir la tasa de abandonos que puede adoptarse es que los participantes que no asistan a la evaluación posterior a la intervención serán llamados de nuevo en la evaluación de seguimiento para evitar la pérdida de datos. En el supuesto de que existan datos faltantes se implementarán imputaciones múltiples mediante ecuaciones encadenadas.

**Cronograma**

El estudio se realizará durante los tres años siguientes a la obtención del permiso por parte del Comité de Ética.

Durante el primer año se planificarán tareas que incluyen la puesta en marcha del estudio de viabilidad y el estudio piloto, así como reuniones del equipo investigador para la puesta en común, discusión y seguimiento del proyecto.

Durante el segundo año se procederá al reclutamiento de los participantes del estudio de investigación, a su aleatorización en diferentes grupos y la implementación de las intervenciones basadas en mindfulness correspondientes. Además, se llevará a cabo el proceso de evaluación cuantitativa y cualitativa y se mantendrán reuniones de equipo para la puesta en común, discusión y seguimiento de los resultados.

Durante el tercer año se llevará a cabo la depuración de la base de datos resultante y su correspondiente análisis para dar respuesta a los objetivos de estudio propuestos. Asimismo, se prepararán y presentarán diversas comunicaciones con los resultados de estudio en diferentes foros científicos (congresos, jornadas, eventos de divulgación…) y no científicos. Además, se llevará a cabo la redacción de dos manuscritos con los resultados del estudio y su posterior publicación en revistas de alto factor de impacto en el JCR. Como en los años anteriores, se mantendrán reuniones de equipo para la puesta en común, discusión y seguimiento de los resultados.

| 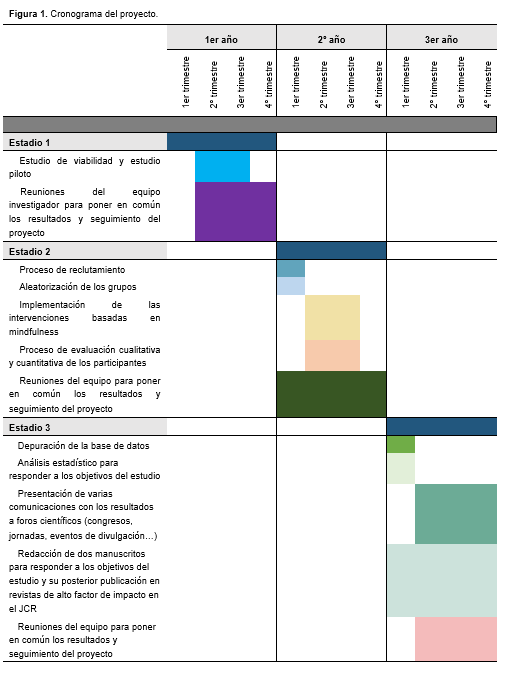 |
| --- |

**Referencias bibliográficas**

1. Sheldon E, Simmonds-Buckley M, Bone C, Mascarenhas T, Chan N, Wincott M, et al. Prevalence and risk factors for mental health problems in university undergraduate students: A systematic review with meta-analysis. J Affect Disord. 2021;287:282-92.

2. Brown P. The invisible problem? Improving students’ mental health. :66.

3. Yusoff MSB, Abdul Rahim AF, Yaacob MJ. Prevalence and Sources of Stress among University Sains Malaysia Medical Students. Malays J Med Sci MJMS. 2010;17(1):30-7.

4. Yf J, Yt L. PREVALENCE AND DETERMINANTS OF PERCEIVED STRESS AMONG UNDERGRADUATE STUDENTS IN A MALAYSIAN UNIVERSITY. J Health Transl Med [Internet]. 2018 [citado 7 de diciembre de 2022];21(1). Disponible en: https://jummec.um.edu.my/index.php/jummec/article/view/11016

5. Modrego-Alarcón M, López-Del-Hoyo Y, García-Campayo J, Pérez-Aranda A, Navarro-

Gil M, Beltrán-Ruiz M, et al. Efficacy of a mindfulness-based programme with and

without virtual reality support to reduce stress in university students: A randomized

controlled trial. Behav Res Ther. 2021; 142:103866.

6. Sharp J, Theiler S. A Review of Psychological Distress Among University Students:

Pervasiveness, Implications and Potential Points of Intervention. Int J Adv Couns.

2018;40(3):193-212.

7. Auerbach RP, Alonso J, Axinn WG, Cuijpers P, Ebert DD, Green JG, et al. Mental

disorders among college students in the World Health Organization World Mental

Health Surveys. Psychol Med. 2016;46(14):2955-70.

8. Deng J, Zhou F, Hou W, Silver Z, Wong CY, Chang O, et al. The prevalence of depressive

symptoms, anxiety symptoms and sleep disturbance in higher education students

during the COVID-19 pandemic: A systematic review and meta-analysis. Psychiatry

Res. 2021; 301:113863.

9. Gómez PP. Equilibrio ocupacional en estudiantes de terapia ocupacional [Internet]

[http://purl.org/dc/dcmitype/Text]. Universidad Miguel Hernández; 2017 [citado 3

de marzo de 2021]. Disponible en:

https://dialnet.unirioja.es/servlet/tesis?codigo=109869

10.Dhas BN, Wagman P. Occupational balance from a clinical perspective. Scand J Occup

Ther. 2022;29(5):373-9.

11.Demiryi M, Berezin S, Fernández G, Ganso H, Gaiteiro A. Primera etapa del proyecto

de investigación. Las rutinas diarias y el balance ocupacional de los alumnos de la

Universidad Nacional de Quilmes.. Rev Chil Ter Ocupacional.

2013;13(2):.g. 73-78.

12.Romero-T.bar A, Rodríguez-Hernández M, Segura-Fragoso A, Cantero-Garlito PA.

Analysis of Occupational Balance and Its Relation to Problematic Internet Use in

University Occupational Therapy Students. Healthc Basel Switz.

2021;9(2):197.

13.Regehr C, Glancy D, Pitts A. Interventions to reduce stress in university students: a

review and meta-analysis. J Affect Disord. 2013;148(1):1-11.

14.Levit-Binnun N, Arbel K, Dorjee D. The Mindfulness Map: A Practical Classification

Framework of Mindfulness Practices, Associated Intentions, and Experiential

Understandings. Front Psychol. 2021;12:727857.

15.Shapiro SL, Carlson LE, Astin JA, Freedman B. Mechanisms of mindfulness. J Clin

Psychol. 2006;62(3):373-86.

16.Baer RA. Mindfulness training as a clinical intervention: A conceptual and empirical

review. Clin Psychol Sci Pract. 2003;10:125-43.

17.Brown KW, Ryan RM, Creswell JD. Mindfulness: Theoretical foundations and

evidence for its salutary effects. Psychol Inq. 2007;18:211-37.

18.Bishop SR. What Do We Really Know About Mindfulness-Based Stress Reduction?

Psychosom Med. 2002;64(1):71-83.

19.Kabat-Zinn J. Full catastrophe living: Using the wisdom of your body and mind to face

stress, pain and illness. New York, NY: Delacorte; 1990.

20.Kabat-Zinn J. Wherever You Go, There You Are: Mindfulness Meditation in Everyday

Life. Hachette Books; 1994. 132 p.

21.Dawson AF, Brown WW, Anderson J, Datta B, Donald JN, Hong K, et al. Mindfulness-

Based Interventions for University Students: A Systematic Review and Meta-Analysis

of Randomised Controlled Trials. Appl Psychol Health Well-Being.

2020;12(2):384-410.

22.Pedrelli P, Nyer M, Yeung A, Zulauf C, Wilens T. College Students: Mental Health

Problems and Treatment Considerations. Acad Psychiatry J Am Assoc Dir Psychiatr

Resid Train Assoc Acad Psychiatry. 2015;39(5):503-11.

23.Furlong J, Davies C. Young people, new technologies and learning at home: Taking

context seriously. Oxf Rev Educ. 2012;38:45-62.

24.Uschner D, Schindler D, Hilgers RD, Heussen N. randomizeR: An R Package for the

Assessment and Implementation of Randomization in Clinical Trials. J Stat Softw.

2018;85(8):1-22.

25.Rosenberg M. Comunicación no violenta: un lenguaje de vida. Acanto; 2016. 276 p.

26. Rogers CR. Grupos de encuentro. Amorrortu; 2018. 192 p.16

27.S.nchez-P.rez A, Mendialdua-Canales D, Hurtado-Pomares M, Peral-Gómez P,

Juárez-Leal I, Espinosa-Sempere C, et al. The ATENción Plena en Enfermedad de

Alzheimer (ATENEA-Mindfulness in Alzheimer’s Disease) Program for Caregivers:

Study Protocol for a Randomized Controlled Trial. Healthc Basel Switz.2022;10(3):542.

28.Bonilla-Jimenez FI, Escobar J. Grupos focales: una guía conceptual y metodológica. 2017 [citado 7 de diciembre de 2022]; Disponible en:

http://148.202.167.116:8080/xmlui/handle/123456789/957

29.Corbin J, Strauss A. Basics of Qualitative Research: Techniques and Procedures for

Developing Grounded Theory. SAGE Publications; 2014. 457 p.
